# Clinical severity score-guided metagenomic analysis identifying gut microbial taxonomic and functional markers for severe hepatitis E progression

**DOI:** 10.1128/msystems.00956-26

**Published:** 2026-09-25

**Authors:** Haiyan Shi, Yanfei Chen, Xiaoli Liu, Jiezuan Yang, Dong Yan, Hua Zhang, Jun Chen, Zhengjie Wu, Danying Yuan, Haifeng Lu, Lanjuan Li

**Affiliations:** 1 State Key Laboratory for Diagnosis and Treatment of Infectious Diseases, National Clinical Research Center for Infectious Diseases, China-Singapore Belt and Road Joint Laboratory on Infection Research and Drug Development, National Medical Center for Infectious Diseases, Collaborative Innovation Center for Diagnosis and Treatment of Infectious Diseases, The First Affiliated Hospital, Zhejiang University School of Medicine 26441, Hangzhou, Zhejiang, China; 2 Department of Infectious Diseases, Ninghai First Hospital 71069, Ninghai, Zhejiang, China; 3 Yuhang Institute of Medical Science Innovation and Transformation, Hangzhou, Zhejiang, China; Istanbul Medipol Universitesi Uluslararasi Tip Fakultesi, Istanbul, Türkiye

**Keywords:** hepatitis type E, XGBoost, LASSO, *Veillonella*, *Streptococcus*

## Abstract

Although gut microbiome alterations have been reported in hepatitis E (HE), the taxonomic and functional determinants of disease severity remain poorly defined. Here, we performed shotgun metagenomic sequencing of fecal samples from 125 individuals spanning acute non-icteric hepatitis (ANIH), acute icteric hepatitis (AIH), acute liver failure (ALF), and healthy controls. We constructed a **s**urrogate clinical severity index from routine blood parameters and developed an integrative analytical framework that combined XGBoost-based taxonomic modeling with LASSO-driven functional feature selection to explore microbiome-severity associations. The taxonomic model discriminated patients from controls (area under the curve [AUC] = 0.944) and identified nine bacterial species significantly associated with severity, independent of age, sex, and body mass index (BMI) (permutation test, *P* < 0.05), with *Veillonella atypica* emerging as the most robust candidate biomarker. The signature featured enrichment of lactate-utilizing *Veillonella* spp. and *Ligilactobacillus salivarius*, alongside depletion of beneficial commensals (*Dorea longicatena*, *Ruminococcus timonensis*, *Eubacterium ramulus*), collectively suggesting a pathogenic “lactate axis.” The HE-enriched lactate utilizers were positively associated with 13 core severity-increasing KOs involved in oxidative stress adaptation (*npr*, *hemQ*, *NUDT1*, *nfr1*) and secretion/biofilm formation (*gspD*, *fhaC*, *vpr*, *sinR*) and negatively with three severity-decreasing KOs, including the butyrate fermentation gene (K14534). Strikingly, the stringent 16-KO core (intersection of four methods) explained more variance in disease severity than the broader set identified by at least three methods (*R*² = 0.61 vs. 0.47). Collectively, these findings reveal a strong link between microbiome-derived lactate metabolism and HE severity, highlighting its potential as a basis for microbiome-based severity stratification and motivating further mechanistic exploration.

**IMPORTANCE:**

Hepatitis E virus (HEV) infection ranges from ANIH to ALF, yet the role of the gut microbiome remains poorly understood across the severity spectrum. By performing shotgun metagenomic sequencing on fecal samples from 125 individuals and applying an integrative framework combining XGBoost-based taxonomic modeling with LASSO-driven functional selection, we identified robust microbiome–severity associations as quantified by a surrogate clinical severity index derived from routine blood parameters. These associations were characterized by enrichment of lactate-utilizing *Veillonella* species (notably *Veillonella atypica*), depletion of beneficial butyrate-producing commensals, and severity-linked shifts in microbial functional gene profiles. Such patterns are consistent with a perturbed microbial “lactate axis” in gut–liver crosstalk, although all associations remain correlational. Our findings nominate candidate microbial markers for severity stratification in hepatitis E and provide a hypothesis-generating framework to guide future mechanistic studies and microbiome-based therapeutic strategies.

## INTRODUCTION

Hepatitis E (HE) caused by HE virus (HEV) infection—a globally prevalent pathogen—is transmitted primarily via the fecal−oral route, as well as through blood transfusion and vertical transmission (1). Historically regarded as self-limited acute hepatitis, HE has since been recognized to exhibit a more complex clinical spectrum (2). Disease progression is influenced by multiple factors, including viral genotype—with genotypes 1 and 2 being associated with large outbreaks and a higher risk of fulminant hepatitis in endemic regions—as well as host immune status (3). Emerging evidence also suggests that the gut microbiome, which co-evolves with the host, may serve as a key modulator of disease outcomes in viral hepatitis infections (4, 5). Mechanistically, the gut microbiome shapes antiviral immunity through pattern recognition receptors, for example, toll-like receptors and nucleotide-binding oligomerization domain-like receptors, (6) and compositional shifts, such as *Bacteroides fragilis* abundance, have been shown to correlate with disease severity in acute HE (5). Furthermore, the co-evolutionary interplay between gut microbiota and host via the gut–liver axis critically influences viral hepatitis outcomes (4, 7). Nevertheless, the specific microbial functional drivers underlying HE progression remain insufficiently understood, and effective prognostic biomarkers or therapeutic targets are urgently needed.

Furthermore, gut dysbiosis is increasingly linked to severe liver disease progression, yet the specific microbial functional genes and their coordinated shifts remain poorly understood. Patients with chronic hepatitis exhibit distinct microbial profiles compared with healthy individuals, and the degree of liver insufficiency correlates closely with dysbiosis severity (8). Mechanistically, gut microbial products—particularly lipopolysaccharide—are detected by pattern recognition receptors in the portal circulation, triggering the production of pro-inflammatory cytokines that amplify hepatic inflammation and escalate disease progression (9). Similar patterns of dysbiosis characterized by expansion of opportunistic pathogens (e.g., *Enterobacteriaceae*, *Neisseria*, and *Gemella*) and depletion of beneficial commensals, including butyrate- and lactate-producing species, have been documented across various liver diseases, including cirrhosis, alcoholic hepatitis, and non-alcoholic fatty liver disease, and are consistently linked to progressive liver injury (10, 11). Collectively, the gut–liver axis as a critical determinant of disease outcomes and provide a rationale for identifying microbial functional drivers that accompany progression to severe HE. Nevertheless, whether a distinct functional reprogramming of the gut microbiome accompanies the transition from acute HE to severe or fulminant disease—and the specific microbial taxa and functional genes involved—remain largely unexplored.

To address this gap, we performed shotgun metagenomic sequencing on a well-characterized cohort comprising patients with acute HE, including acute-on-chronic liver failure, icteric, and non-icteric cases, and healthy controls (HC). We first constructed a composite clinical score to objectively quantify disease severity, integrating key biochemical and hematological parameters through trend tests and ordinal logistic regression with bootstrap cross-validation. This score was developed as a surrogate index for clinical severity—a dimensionality-reduction tool that captures overall disease burden rather than a novel biological phenotype—to ensure robust quantification across the clinical spectrum. Subsequently, using this score, we employed XGBoost to develop classification and regression models based on taxonomic profiles. Finally, we applied four complementary feature selection methods (Spearman, Kendall, bootstrap, and least absolute shrinkage and selection operator [LASSO]) to identify microbial functional genes robustly associated with the clinical score. Integrating these analyses, we uncovered coordinated shifts in gut microbial composition and function that accompany the development of severe HE.

## MATERIALS AND METHODS

### Participant information

The study was approved by the Ethics Committee of the First Affiliated Hospital, School of Medicine, Zhejiang University (reference number: IIT20230384B-R3). All procedures were conducted in accordance with the ethical guidelines of the 1975 Declaration of Helsinki. Written informed consent was obtained from all participants.

From December 2022 to May 2025, a total of 81 eligible participants were enrolled. The inclusion criteria were as follows: (i) patients aged 45–70 years with confirmed HEV infection (HEV-IgM positive) were enrolled; this age range captures the high-risk demographic for severe hepatitis E while excluding younger patients with distinct etiologies and elderly patients with comorbidities or polypharmacy; (ii) with virologically confirmed HEV infection; (iii) reporting liver injury without other identifiable causes; and (iv) Han Chinese, Zhejiang residents with similar dietary and lifestyle habits. The exclusion criteria were as follows: (i) those with non-HEV hepatitis virus infection; (ii) reporting pregnancy or immunocompromised state; (iii) having other liver diseases or drug-induced injury; (iv) reporting excessive substance use; (v) reporting antibiotic use within 6 weeks; (vi) reporting major surgery within 5 years; or (vii) with chronic nutritional, metabolic, or immunological disorders. Based on disease severity and the presence of jaundice, HEV-IgM-positive participants were classified into three groups: (i) acute liver failure (ALF) group (*n* = 28), comprising patients with severe liver impairment who progressed to grades I–IV hepatic encephalopathy within 2 weeks, accompanied by gastrointestinal symptoms (anorexia, abdominal distension, nausea, vomiting), progressively deepening jaundice, serum total bilirubin increase ≥17.1 µmol/L per day, prothrombin activity ≤40%, or international normalized ratio ≥1.5 (12); (ii) acute icteric HE (AIH) group (*n* = 38), comprising patients presenting with dark urine and clinical jaundice; and (iii) acute non-icteric HE (ANIH) group (*n* = 15), comprising patients with acute hepatitis and mild liver impairment (abnormal ALT/AST) but without clinical jaundice. Patients were restricted to 45–70 years, reflecting the high-risk demographic for hepatitis E and severe disease while minimizing confounding from younger patients with distinct etiologies and elderly patients with comorbidities or polypharmacy.

Furthermore, 44 age-, sex-, and body mass index (BMI)-matched HC were enrolled based on routine examination findings. Controls had no chronic diseases and were not taking any chronic medications, including proton pump inhibitors, within 3 months prior to enrollment. Data were collected on demographics (age, sex, BMI), clinical parameters (from blood routine, biochemical, and hepatitis virus testing, shown in Table S1a), and dietary and lifestyle habits. Dietary assessment confirmed that all participants followed a conventional Zhejiang diet defined by rice-centered meals with vegetable and meat side dishes.

### Specimen collection and DNA extraction

Well-formed feces and three blood samples (two plain tubes and one EDTA-containing tube) were collected from each participant following our previously described protocol (13). All samples were collected either at the outpatient clinic or on the first day of hospital admission before any medical interventions had been initiated. Briefly, stool specimens were aliquoted into 200–220 mg portions in the laboratory and refrigerated at −80°C within 1 h. Blood samples were labeled and immediately delivered to the hospital’s clinical diagnostics laboratory for routine blood tests, biochemical parameter analysis, and hepatitis viral measurements using standardized protocols; no multiple instruments or interviewers were involved. Total DNA was extracted in our lab from all stool samples using the QIAGEN DNeasy PowerSoil Kit (cat. no. 12888, Hilden, Germany), quantified with the Qubit dsDNA HS Assay Kit, and normalized to 20 ng/μL for sequencing library preparation, as detailed in our previous study (13).

### Metagenomic sequencing and bioinformatic analysis

#### Shotgun metagenomic sequencing

Shotgun metagenomic sequencing was performed in our lab following the protocol described in our previous study (13). Briefly, 10 μL of DNA per sample was fragmented by sonication to achieve a target insert size of 400–550 bp. Sequencing libraries were constructed using the NEBNext Ultra DNA Library Prep Kit for Illumina (NEB, USA), whereby unique index adapters were ligated to each sample for multiplexing. Library quality was assessed using the Qubit 2.0 fluorometer (Life Technologies, CA, USA) and Agilent Bioanalyzer 2100 System. Paired-end sequencing (2 × 150 bp) was conducted on the Illumina NovaSeq 6000 platform. The raw sequencing data have been deposited in the National Microbiology Data Center under accession numbers PRJNA1188794 and 1449747. Quality controls are detailed in File S2. Briefly, a custom positive control (*B. longum* and *E. coli*) and a negative control (sterile water) were included in each sequencing batch to monitor extraction efficiency, library preparation, and contamination. The positive control consistently yielded the expected composition, and no significant microbial reads were detected in the negative controls.

#### Quality control, assembly, and binning

The raw paired-end reads were first quality-trimmed using Fastp (v0.23.0) (14) to remove adapter sequences, ambiguous bases, and low-quality reads (average *Q*-score < 20). We decontaminated a human gut microbiome with short reads using HoCoRT (15) by aligning reads against the human reference genome (GRCh38). The cleaned reads were assembled into contigs using MEGAHIT (16). Subsequently, MetaBAT2 (17) was employed to bin the assembled contigs into metagenome-assembled genomes (MAGs) based on tetranucleotide frequency and abundance profiles. The completeness and contamination of the resulting MAGs were evaluated with CheckM (18) to retain high-quality MAGs (completeness >70% and contamination <10%), and only high-quality MAGs were retained for downstream analyses.

#### Taxonomic profiling

To profile species-level taxonomic abundances, we developed a custom database of universal single-copy genes (USCGs) derived from OrthoDB (the hierarchical catalog of orthologs; http://www.orthodb.org) (19). Clean reads were aligned to this USCG database using minimap2 (20), and reads per kilobase per million values were calculated for each taxon per sample to generate the microbial taxonomic abundance table. The custom analysis scripts are available on GitHub (Zhou-lab-SUDA/ucgMLST).

#### Gene prediction and functional annotation

Gene prediction and functional annotation were performed on high-quality MAGs. Prokka (21) was used for initial gene calling, and functional annotations were assigned by querying against the Kyoto Encyclopedia of Genes and Genomes (KEGG) (22, 23), the Carbohydrate-Active Enzyme Database (24), and the Comprehensive Antibiotic Resistance Database (25) using an *E*-value cutoff of 1 × 10⁻⁵.

#### Generation of stratified functional profiles

The HUMAnN 3.0 pathway profiling tool (26) was used to generate the final stratified pathway abundance table (pathabundance_stratified.tsv). The reads were first mapped to the combined set of predicted gene sequences from the MAGs. For each gene, read counts were aggregated at both the KEGG Orthology (KO) level and taxonomic level (derived from USCG-based assignments). The resulting abundances for each pathway were normalized to transcripts per million and reported per species, thereby providing a stratified functional profile.

More detailed information regarding the preprocessing steps, quality metrics, and batch correction procedures is provided in the Supplemental file.

### Development of a surrogate index for clinical severity

To quantitatively assess disease severity, we developed a composite scoring system based on routine biochemical and hematological parameters. First, the Jonckheere–Terpstra trend test was applied to all candidate parameters to identify those with monotonic trends across ordered disease groups (HC, ANIH, AIH, ALF), with FDR correction (*q* < 0.05) and |Spearman ρ| >0.75 as selection criteria. Age, sex, and BMI were included as a priori covariates in all ordinal logistic regression models to control for confounding.

All possible two- to five-parameter combinations of the qualifying candidates were evaluated via 100× bootstrap resampling. For each combination, three metrics were assessed: (i) Akaike information criterion (AIC) for model fit; (ii) Spearman ρ between predicted score and disease severity; and (iii) bootstrap success rate, defined as the percentage of resamples yielding a converged model with directionally consistent ρ. These metrics were aggregated into a panel selection score using a weighted linear combination (wρ = 0.35, wBS = 0.35, wAIC = 0.30), where the normalized AIC component was defined as 1 − (AIC − min_AIC)/(max_AIC − min_AIC). The combination with the highest panel selection score was selected as the optimal panel. To ensure robustness, we performed a weight sensitivity analysis by varying the weights across 10 configurations. The selected panel ranked first or second in 60% of scenarios and first under the original weights, confirming selection stability (Table S2a).

The final clinical severity score (hereinafter referred to as Final_Score) was computed as the weighted sum of standardized parameters (*Z*-scores) and scaled to 0–100. To avoid circularity, feature importance (Gain) and Spearman |ρ| were calculated strictly within each bootstrap training set; hybrid weights were applied to out-of-bag samples for evaluation. Model performance was assessed by fivefold stratified cross-validation, reporting *R*², adjusted *R*², RMSE, and MAE. To rule out demographic confounding of the Final_Score, we compared three predictive models: (i) confounders only (age, sex, BMI); (ii) clinical indicators only (AST, PT, TBA, CHE); and (iii) clinical indicators plus confounders. The incremental predictive value of confounders was evaluated by comparing adjusted *R*², AIC, and Spearman ρ across models.

### Microbiome-based statistical and machine learning analyses

#### Data preprocessing

Species-level microbial abundance data (Table S1b) were filtered to retain only species detected in ≥10% of samples. To reduce noise, species with a coefficient of variation <0.5 among positive samples were excluded. The remaining abundances were transformed using centered log ratio (CLR) after adding a pseudocount of 1. The clinical severity score was normalized to zero mean and unit variance.

### Classification analysis: distinguishing patients from HCs

To distinguish patients from healthy controls based on gut microbiome profiles, we employed an extreme gradient boosting (XGBoost) classifier using the xgboost R package (v1.7.5). The binary response was patient (ANIH, AIH, ALF) versus healthy control (HC), with input features comprising exclusively CLR-transformed species-level abundances.

#### Independent predictive value of microbiome beyond demographic confounders

To comprehensively assess the discriminative capacity of microbiome and demographic variables, we constructed and compared three independent XGBoost classifiers across 100 bootstrap iterations: (i) confounders only (age, sex, BMI); (ii) microbiome only; and (iii) microbiome + confounders. Each model was trained on bootstrap samples (with replacement) and evaluated on out-of-bag (OOB) samples. The independent predictive value of microbiome data was assessed by comparing AUC distributions: microbiome only versus confounders only to evaluate microbiome’s discriminative capacity independent of demographics. Additionally, a permutation test (*n* = 100) was performed on an independent 80/20 train-test split: microbial features were permuted, while confounders were held constant, and the null distribution of ΔAUC (microbiome + confounders vs. confounders only) was used to calculate a permutation *P*-value.

#### Threshold selection and stability validation

A comprehensive grid search (range, 0.1–0.9; step, 0.05) was performed on the consensus probabilities from the microbiome-only model. At each candidate threshold, we evaluated sensitivity, specificity, accuracy, precision, and the Youden index. The operational threshold was selected by jointly considering these metrics, with priority given to higher sensitivity at the cost of modestly lower specificity, reflecting the clinical screening context where minimizing missed diagnoses is prioritized. To assess robustness, the Youden-optimal cutoff was independently recalculated within each bootstrap iteration using only OOB predictions. The bootstrap-derived threshold distribution (mean, SD, CV, 95% CI) was summarized to evaluate whether the selected operational threshold lies within the confidence region of the data-driven optimum.

#### Feature importance and robustness

Feature importance was quantified using the Gain metric from the final microbiome-only XGBoost model. To evaluate rank stability across the 100 bootstrap iterations, species absent from a given iteration’s importance list were assigned the lowest possible rank (max rank + 1). For each species, we calculated the mean rank, SD, 95% CI, and presence rate (frequency of appearance across iterations), ensuring that inconsistently important features were appropriately penalized.

#### Per-species permutation test for statistical significance

To determine whether individual species importance was statistically robust rather than driven by random covariation, we performed a per-species permutation test on the microbiome-only model. The entire CLR-transformed microbiome abundance matrix was globally permuted by random shuffling of sample indices, decoupling microbial features from disease status while preserving the marginal distribution of each species. For each of 100 permutations, an XGBoost model was retrained on the permuted data, and feature importance was quantified by Gain. This generated a null distribution of Gain values for each species under the hypothesis of no association with disease. The per-species significance was assessed by comparing the true Gain from the original (unpermuted) model against its permutation-derived null distribution; one-sided *P*-value was computed as the proportion of permuted Gain values equal to or exceeding the observed true Gain. *P*-values were adjusted using the Benjamini–Hochberg false discovery rate (FDR) procedure, and species with FDR <0.05 were deemed statistically significant.

#### Confounder-adjusted sensitivity analysis

To rule out the possibility that feature importance rankings were driven by demographic differences between groups, we performed a confounder-adjusted validation. For each species, we fitted a linear regression of CLR-transformed abundance against age, sex, and BMI, extracted the residuals, and re-trained the XGBoost classifier on the residualized abundance matrix. Feature importance rankings from the original and confounder-adjusted models were compared; species with |_Δ_Rank| ≤ 5 were classified as robust; those with 5 < |_Δ_Rank| ≤ 15 were classified as moderate; and those with |_Δ_Rank| > 15 were classified as sensitive. The mean variance in species abundance explained by confounders (*R*²) was also quantified.

All reported classification performance metrics (accuracy, sensitivity, specificity, and AUC) are based on consensus predictions aggregated across bootstrap iterations, with 95% confidence intervals derived from the bootstrap distribution.

### Regression analysis: identifying microbes associated with disease severity

To identify bacterial species whose abundance correlated with disease severity, we employed XGBoost regression using the clinical severity score (Final_Score) as the response variable. The model input comprised CLR-transformed species abundances, with age, sex, and BMI included as covariates to control for confounding.

Model performance was evaluated using fivefold cross-validation and 100 bootstrap iterations, with cross-validated *R*², RMSE, and Spearman correlation as metrics. In each iteration, a bootstrap sample (with replacement) was drawn; an XGBoost model was trained; and predictions were generated on the out-of-bag samples. Feature importance was quantified by the Gain metric, with mean and SD computed across bootstrap iterations.

#### Per-species permutation test for feature importance

To determine which individual species contributed significantly to the microbiome-only model beyond random noise, we conducted a per-species permutation test within the XGBoost regression framework (839 CLR-transformed species + age/sex/BMI as covariates). In each of 100 permutations, the rows of the microbial feature matrix were randomly shuffled, while covariates were held constant. An XGBoost model was trained on the permuted data, and the Gain importance was extracted for each species. The permutation *P*-value was calculated as the proportion of permutations in which the Gain under the null (permuted) distribution equaled or exceeded the true Gain observed in the original data. Species with permutation *P* <0.05 were considered to exhibit significant importance independent of random covariation.

#### Composite scoring for species importance

We evaluated each species across three complementary dimensions: (i) XGBoost Gain (mean across 100 bootstrap iterations), reflecting the model’s reliance on the species for predicting the clinical severity score; (ii) absolute Spearman correlation with the continuous Final_Score, capturing linear abundance changes across the severity spectrum; and (iii) absolute Spearman correlation with the ordinal disease group variable (HC < ANIH < AIH < ALF), reflecting monotonic trends across clinically defined stages. *P*-values for both correlation metrics were FDR-adjusted, and species with adjusted *P* <0.05 were considered to exhibit significant monotonic trends. Each metric was first standardized to zero mean and unit variance at the species level. To derive sample-level scores, we calculated abundance-weighted averages of the corresponding standardized species-level metrics across all species using raw species abundance as weights. The relative contribution (weight) of each dimension was then determined by a multivariate ordinal logistic regression of disease group on the three sample-level scores; the absolute regression coefficients were normalized to sum to 1. For each species, the standardized values of the three metrics were multiplied by the corresponding weights and summed to obtain the composite score. To evaluate the stability of these weights against training set variability, we performed fivefold cross-validation: in each fold, the three weight components were re-estimated using the same ordinal logistic regression framework on the training set, and the distributions of the weights across folds were examined.

#### Core species definition

The core species were defined as those simultaneously ranked in the top 50 by the composite score and achieving statistical significance (*P* < 0.05) in the per-species permutation test. To compare the relative abundances of the core species across disease severity groups (HC, ANIH, AIH, ALF), Kruskal−Wallis tests were performed on CLR-transformed relative abundances (with a pseudocount of 1 added prior to transformation). Prevalence (percentage of samples with non-zero abundance per group) was compared across groups using χ tests (with simulated *P*-values when expected cell counts < 5). All statistical analyses were conducted in R (version 4.5.1) using the tidyverse, rstatix, FSA, and ggplot2 packages.

### Feature selection for identifying robust microbial functional markers

To identify microbial functional genes (KEGG orthologs, Kos; Table S1d) robustly associated with disease severity, we applied four complementary feature selection methods: Spearman correlation, Kendall correlation, bootstrap resampling, and LASSO regression. The final set of core KOs was defined as the intersection of KOs selected by all four methods. All analyses were performed in R (version 4.5.1). The clinical severity score (Final_Score) was residualized for confounders (age, gender, and BMI) via linear regression, and the residuals were used as the adjusted outcome variable for all downstream analyses.

#### Spearman rank correlation

For each KO, Spearman’s rank correlation coefficient (ρ) with the residualized clinical score was calculated using the cor.test function (base R). KOs with |ρ| ≥ 0.3 and FDR < 0.05 (Benjamini–Hochberg method, *p*.adjust function) were retained.

#### Kendall’s *τ* rank correlation

Kendall’s τ coefficient was computed using the cor.test function (base R) with the same significance thresholds (|*τ*| ≥ 0.3, FDR < 0.05) applied.

#### Bootstrap validation

One hundred bootstrap samples (with replacement), each of the same size as the original data set, were generated. In each iteration, Spearman correlation was recalculated. The 95% bootstrap confidence interval (CI) for ρ was derived from the 2.5th and 97.5th percentiles of the bootstrap distribution. A KO was considered robustly supported if its 95% CI did not overlap the null threshold.

#### LASSO regression

A LASSO model was fitted with the residualized clinical score as the response variable and all KO abundances as predictors using the glmnet package. Fivefold cross-validation was employed to select the optimal regularization parameter λ (lambda.min). KOs with non-zero coefficients in the final model were retained.

#### Sensitivity analysis for feature selection robustness

In response to reviewer concerns that the intersection of all four methods may be overly stringent due to LASSO sparsity and functional collinearity among KOs, we performed a sensitivity analysis defining core KOs as those selected by at least three of the four methods (≥3/4). This threshold was used as the primary feature set for downstream biological interpretation, while the stringent four-method intersection was reported for comparison.

#### KO-to-pathway mapping and enrichment analysis

Pathway enrichment was assessed by hypergeometric tests, where the background gene set comprised all KOs detected in the data set. The enrichment ratio was calculated as the observed-to-expected number of selected KOs per pathway, and FDR correction was applied across all tested pathways.

#### Correlation analysis between key bacterial species and KOs/enriched pathways

Species abundances were CLR-transformed prior to analysis. Spearman’s rank correlation coefficient (ρ) was calculated between each species and each enriched pathway (mean CLR abundance of constituent KOs), followed by FDR correction (Benjamini–Hochberg method). Significant correlations were defined as |ρ|> 0.3 and FDR-adjusted *P* < 0.05. The network graph was constructed using the igraph package (v1.5.1) in R.

### Reporting checklist

The completed STORMS checklist for this study is available at GitHub: https://github.com/Neal050617/Metagenomic_analysis_severe_Hepatitis_E_progression/blob/6d80a436aef656f0397fe8952b832459de78dd98/Supplementary_Table_STORMS_Checklist.pdf.

## RESULTS

### Taxonomic and functional diversity and community composition

At the species level, group differences were characterized primarily by lower richness in AIH and ALF versus HC (Kruskal–Wallis test, FDR < 0.05), along with modest but significant beta diversity separation (PERMANOVA, *R*-squared = 0.079, *P* < 0.001; Fig. S1A; Table S1b and c). The dominant species profiles across groups are presented in Fig. S1B. At the functional gene level, group differences were characterized primarily by higher Shannon index and Pielou’s evenness in all three patient groups (ANIH, AIH, and ALF) versus HC (Kruskal–Wallis test, FDR < 0.01), along with modest but significant beta diversity separation (PERMANOVA, *R*² = 0.101, *P* < 0.001; Fig. S1C; Table S1d and e).

### AST–PT–TBA–CHE panel defining the surrogate index for clinical severity

**Fig 1:**
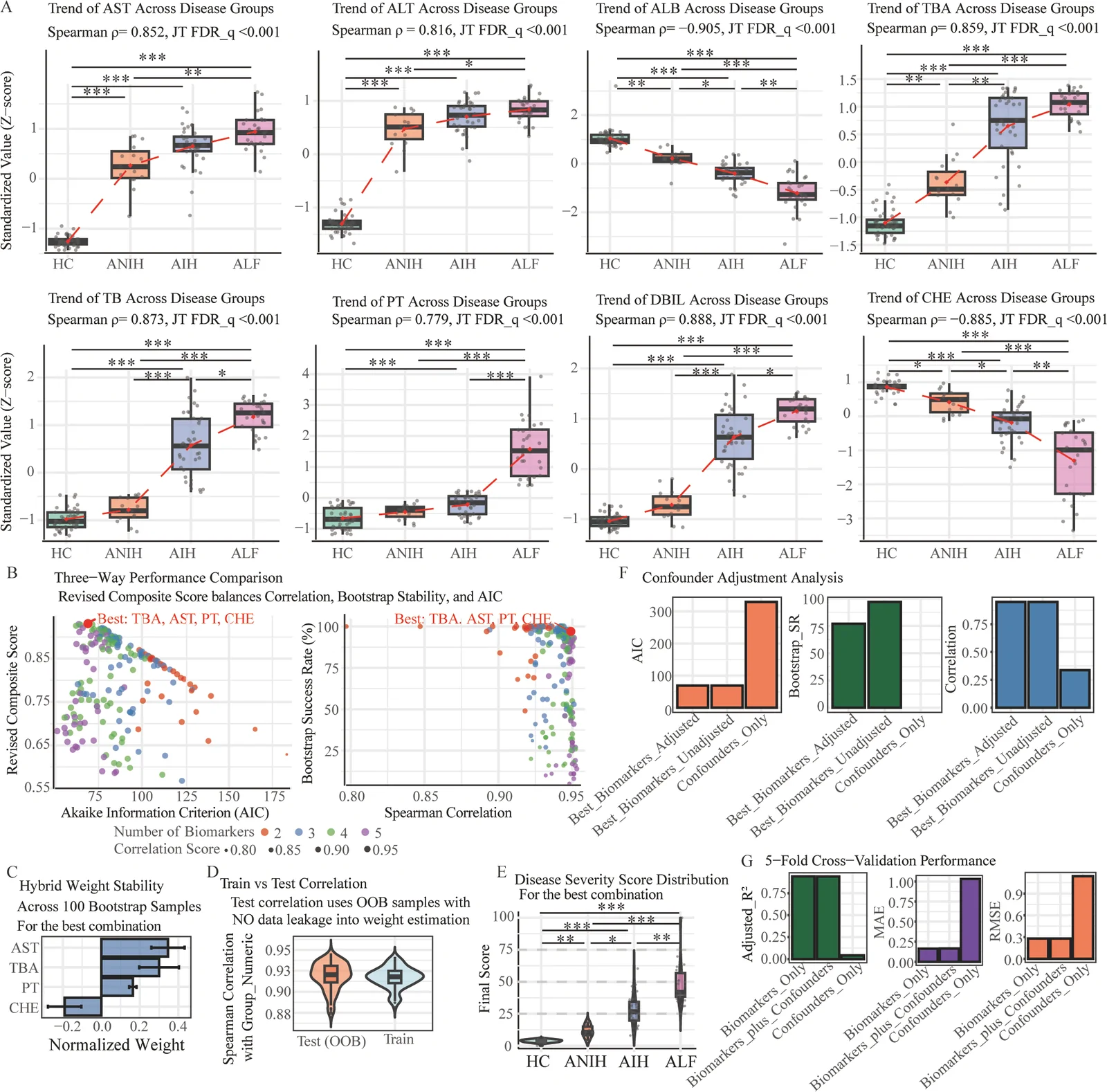
Construction and validation of a composite clinical score for liver disease severity. (**A**) Severity-associated clinical biomarkers across groups (Spearman’s rho and Jonckheere-Terpstra *P* values shown). (**B**) Comparison of 210 biomarker combinations by AIC and bootstrap success rate, with the optimal combination (TBA, AST, PT, and CHE) highlighted in red. (**C**) Hybrid weight stability across 100 bootstrap replicates. (**D**) Train versus out-of-bag Spearman correlations confirming no data leakage. (**E**) Final severity score distribution (0–100 scale). (**F**) Confounder adjustment analysis (unadjusted versus adjusted for age, gender, and BMI). (**G**) Fivefold cross-validation of XGBoost models by adjusted *R*-squared, MAE, and RMSE. Pairwise comparisons in panels **A** and **E**, Wilcoxon rank-sum tests (*P < 0.05, **P < 0.01, and ***P < 0.001). HC, healthy controls; ANIH, acute non-icteric hepatitis; AIH, acute icteric hepatitis; ALF, acute liver failure.

Of those biochemical parameters showing monotonic trends across disease severity groups (Jonckheere–Terpstra test, *q* < 0.05, |Spearman ρ| > 0.75; Fig. 1A, Table S2a), the AST–PT–TBA–CHE panel was selected as the optimal combination based on a composite evaluation of correlation, bootstrap stability, and model fit (Fig. 1B; Table S2b and c). The resulting hybrid weights were 0.39 (AST), 0.30 (TBA), 0.18 (PT), and −0.13 (CHE), and these weights were highly stable across 100 bootstrap replicates (Fig. 1C). Importantly, correlations computed on out-of-bag test samples (median ρ = 0.916) closely matched those of training samples (median ρ = 0.921), confirming that weight estimation was not inflated by data leakage (Fig. 1D). The final score increased stepwise from HC through ANIH and AIH to ALF, with significant differences between all pairwise group comparisons (all FDR < 0.05; Fig. 1E).

Adjusting for age, gender, and BMI did not materially alter model performance (AIC 68.8 vs 68.9; ρ = 0.949 for both), and confounders alone showed little explanatory value (AIC 329.5; ρ = 0.337; Fig. 1F). Fivefold cross-validation confirmed that adding confounders did not improve prediction (adjusted *R*² = 0.911 for both) and that confounders alone performed worse than a null model (Fig. 1G), indicating that the severity score captures disease-relevant information independent of demographic confounders.

### XGBoost classification distinguishing patients from healthy controls

**Fig 2:**
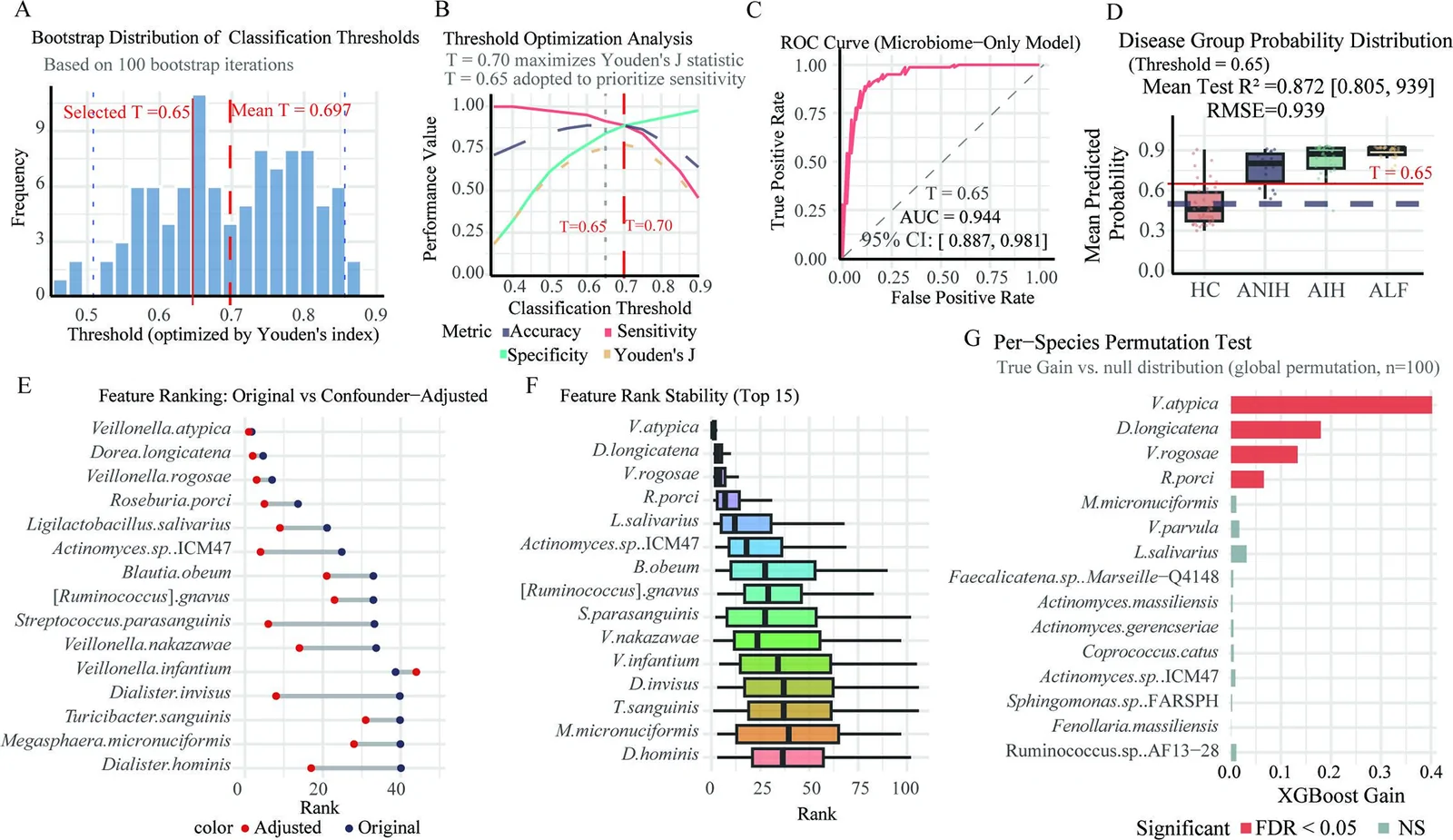
XGBoost classification model for distinguishing patients from healthy controls. (**A**) Bootstrap distribution of optimal thresholds (100 iterations). Red dashed line, mean (0.697); blue dotted lines, 95% CI (0.508–0.856); red solid line, adopted threshold (0.65). (**B**) Threshold optimization. Sensitivity, specificity, and accuracy across thresholds (0.1–0.9; step 0.05). Youden maximizing cutoff = 0.70; operational cutoff = 0.65 (vertical red line), prioritizing sensitivity for screening. (**C**) ROC curve (AUC = 0.944) for the microbiome-only model, with the 0.65 threshold indicated. (**D**) Consensus-predicted probabilities stratified by disease cohort and healthy controls; the red line marks the 0.65 threshold. (**E**) Feature importance ranks of the top 15 species before (dark blue) and after (red) adjustment for age, sex, and BMI; gray lines connect the two ranks of each species. Lower ranks indicate higher importance. (**F**) Rank stability of the top 15 species across 100 bootstrap iterations. Center line, median; box limits, 25th and 75th percentiles; whiskers, 1.5× interquartile range; and species absent from an iteration were assigned the maximum rank + 1. (**G**) Per-species permutation test for the top 15 features. Bars, observed XGBoost Gain; red bars, FDR < 0.05; gray bars, not significant. Four significant species (*V. atypica*, *D. longicatena*, *V. rogosae*, *R. porci*) are highlighted. HC, healthy controls; ANIH, acute non-icteric hepatitis; AIH, acute icteric hepatitis; ALF, acute liver failure; AUC, area under the receiver operating characteristic curve; FDR, false discovery rate.

The microbiome-only XGBoost classifier robustly discriminated patients from healthy controls across 100 bootstrap iterations. The bootstrap-derived optimal classification thresholds were centered at a mean of 0.697 (95% CI: 0.508–0.856) (Fig. 2A). Although the Youden’s *J* statistic was maximized at *T* = 0.70 (*J* = 0.763), we adopted *T* = 0.65 as the operational threshold because it provided higher sensitivity (91.4% vs. 88.9% at *T* = 0.70) and a marginally better F1 score (0.914 vs. 0.911), with a specificity of 84.1%—a more favorable precision–recall balance for patient screening, where minimizing missed diagnoses is the priority (Fig. 2B; Table S3a). This threshold was also among the most frequently selected across bootstrap iterations and fell well within the bootstrap 95% CI, confirming its stability and generalizability. At the adopted threshold, the model achieved an AUC of 0.944 (95% CI: 0.887–0.981) (Fig. 2C). Consensus predicted probabilities rose stepwise from HC through ANIH and AIH to ALF, with nearly all patients above and most controls below the 0.65 cutoff (Fig. 2D; Table S3b), indicating that the model captures a severity-scaled microbiome signature rather than a binary patient–control distinction.

Feature importance analysis identified *Veillonella atypica*, *Dorea longicatena*, *Veillonella rogosae*, and *Roseburia porci* as the top-ranked discriminative species. These rankings were robust to confounding: after residualizing each species' CLR-transformed abundance on age, sex, and BMI, the adjusted ranks closely mirrored the original ranks for nearly all top 15 species (Fig. 2E; Table S3c). The rankings were also highly stable, with the top four species consistently occupying the highest positions across 100 bootstrap iterations (Fig. 2F; Table S3d). Finally, a per-species permutation test (*n* = 100) confirmed that the observed Gain values of *V. atypica*, *D. longicatena*, *V. rogosae*, and *R. porci* significantly exceeded their respective null distributions (FDR < 0.05), while the remaining top features did not reach statistical significance (Fig. 2G; Table S3e). Microbial features raised the AUC of the confounder-only baseline (age, sex, and BMI) from 0.580 to 0.933 (ΔAUC = 0.353, permutation *P* < 0.01), whereas confounders added nothing to the microbiome-only model (mean AUC = 0.932 for both; Fig. S2A and B; Table S3f), indicating that the predictive signal resides almost entirely in the microbial features.

### Key bacteria associated with disease severity

**Fig 3:**
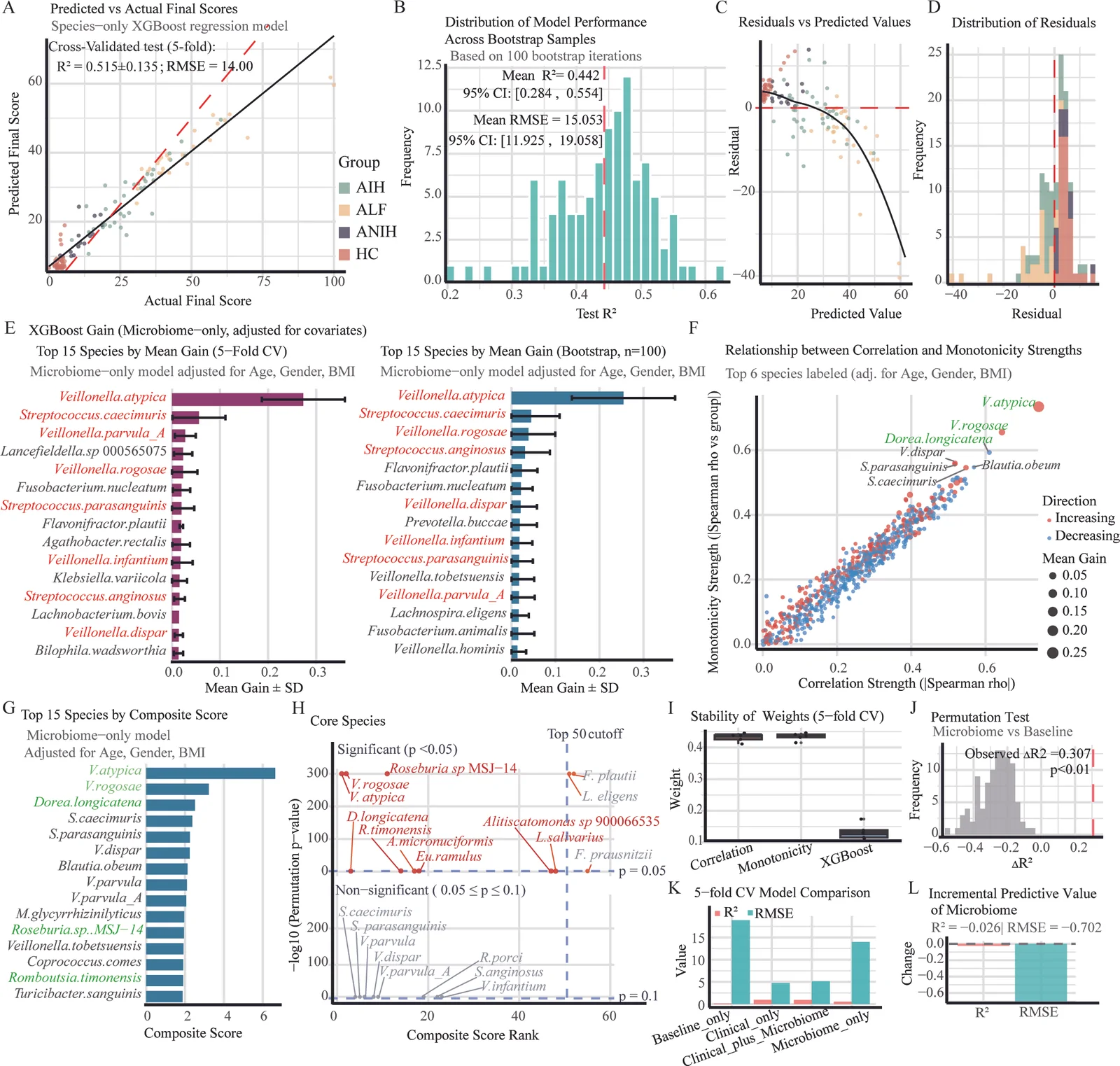
XGBoost regression of the surrogate clinical severity index (final scores) on the gut microbiome. Unless otherwise noted, all models were adjusted for age, gender, and BMI. (**A**) Predicted versus actual final scores (microbiome-only model). (**B**) Test-set *R*-squared distribution across 100 bootstrap iterations (microbiome-only model); red dashed line, mean. (**C, D**) Residuals versus predicted values (**C**) and distribution of residuals (**D**), colored by group. (**E**) Top 15 species by mean XGBoost gain (mean ± SD) across fivefold cross-validation (left) and 100 bootstrap iterations (right). (**F**) Correlation strength (absolute Spearman rho of abundance versus final score) versus monotonicity strength (absolute Spearman rho of abundance versus ordered groups). Point size denotes mean gain; color denotes trend direction (red, increasing; blue, decreasing). Top six species by composite score are labeled. (**G**) Top 15 species by composite score integrating XGBoost gain, correlation, and monotonicity. (**H**) Per-species permutation test (100 permutations). The *x*-axis represents composite score rank; the *y*-axis represents negative log10 (permutation *P* value). Horizontal dashed lines indicate *P* = 0.05 and *P* = 0.1; the vertical dashed line indicates the top-50 rank cutoff. Red points denote significant core species (*P* < 0.05 and ranked within top 50). (**I**) Stability of composite-score weights across five cross-validation folds. (**J**) Permutation test of microbiome incremental value over the covariate-only baseline (100 permutations). Histogram, permuted delta *R*-squared; red dashed line, observed delta *R*-squared = 0.31 (*P* < 0.01). (**K**) Fivefold cross-validation comparison of four models. (**L**) Incremental predictive value of the microbiome over baseline. Unless otherwise noted, all models were adjusted for age, gender, and BMI. HC, healthy controls; ANIH, acute non-icteric hepatitis; AIH, acute icteric hepatitis; ALF, acute liver failure; BMI, body mass index; TBA, total bile acids; AST, aspartate aminotransferase; PT, prothrombin time; CHE, cholinesterase; CV, cross-validation; RMSE, root mean square error.

After adjusting for age, sex, and BMI, the microbiome-only XGBoost regression model using the clinical severity score as the response achieved a fivefold cross-validated *R*² of 0.515 ± 0.135 and an RMSE of 14.00 (Fig. 3A), and bootstrap validation (100 iterations) confirmed a mean *R*² of 0.442 (95% CI: 0.284–0.554; Fig. 3B), with residuals centered on zero (Fig. 3C and D; Table S4a). Ranking by XGBoost gain identified eight species that appeared in the top 15 under both fivefold cross-validation and 100 bootstrap iterations, including *V. atypica*, *Streptococcus caecimuris*, *V. parvula_A*, *V. rogosae*, *S. parasanguinis*, *V. infantium*, *S. anginosus*, and *V. dispar* (Fig. 3E; Table S4b and c). This concordance indicates that the signature is stable and not driven by a particular data partition. This gain-based signature was largely recapitulated by correlation and monotonicity analyses, in which five of the eight species again ranked among the top correlates of the clinical severity score led by *V. atypica* and *V. rogosae* (Fig. 3F). Notably, *Blautia obeum* and *Dorea longicatena* emerged as additional strongly associated taxa. Integrating gain, correlation, and monotonicity into a composite score ranked *V. atypica* first, followed by *V. rogosae*, *D. longicatena*, *S. caecimuris*, *S. parasanguinis*, and *V. dispar* (Fig. 3F; Table S3d). Meanwhile, the component weights underlying this score, derived from ordinal logistic regression, were highly stable across the five CV folds (XGBoost, 0.11–0.17; monotonicity, 0.42–0.45; correlation, 0.41–0.45; Fig. 3I; Table S4f), confirming that the composite ranking was not driven by fold-specific variation in weighting.

A per-species permutation test identified 26 species with significant contributions (Table S4e). However, the *Streptococcus* species prioritized by XGBoost gain (*S. caecimuris*, *S. parasanguinis*, *S. anginosus*) did not survive this test (all *P* > 0.05), suggesting their gain-based importance reflects correlated rather than independent signals. Nine of these species ranked within the composite top 50 (*V. atypica*, *V. rogosae*, *D. longicatena*, *L. salivarius*, *Eu. ramulus*, *R. timonensis*, *Roseburia* sp. MSJ-14, *A. micronuciformis*, and *Alitiscatomonas* sp. 900066535) and were defined as severity-associated core species (Fig. 3H). *V. rogosae* and *V. atypica* differed significantly in all pairwise group comparisons, while *Eu. ramulus*, *Roseburia* sp. MSJ-14, and *R. timonensis* were significant in all but ANIH versus HC (Fig. S3).

Permutation testing showed that the microbiome-only model significantly outperformed the confounders-only baseline (*R*² = 0.552 vs. 0.245; observed Δ*R*² = 0.307; permutation *P* < 0.01; Fig. 3J; Table S4g), confirming that the gut microbiome carries severity-related information independent of age, sex, and BMI. However, the clinical-only model explained most of the variance (*R*² = 0.941), and adding microbial features did not further improve performance (combined *R*² = 0.930; Fig. 3K and L; Table S4h), indicating that this microbial information is largely captured by routine clinical indicators.

### Core microbial functional genes associated with disease severity

**Fig 4:**
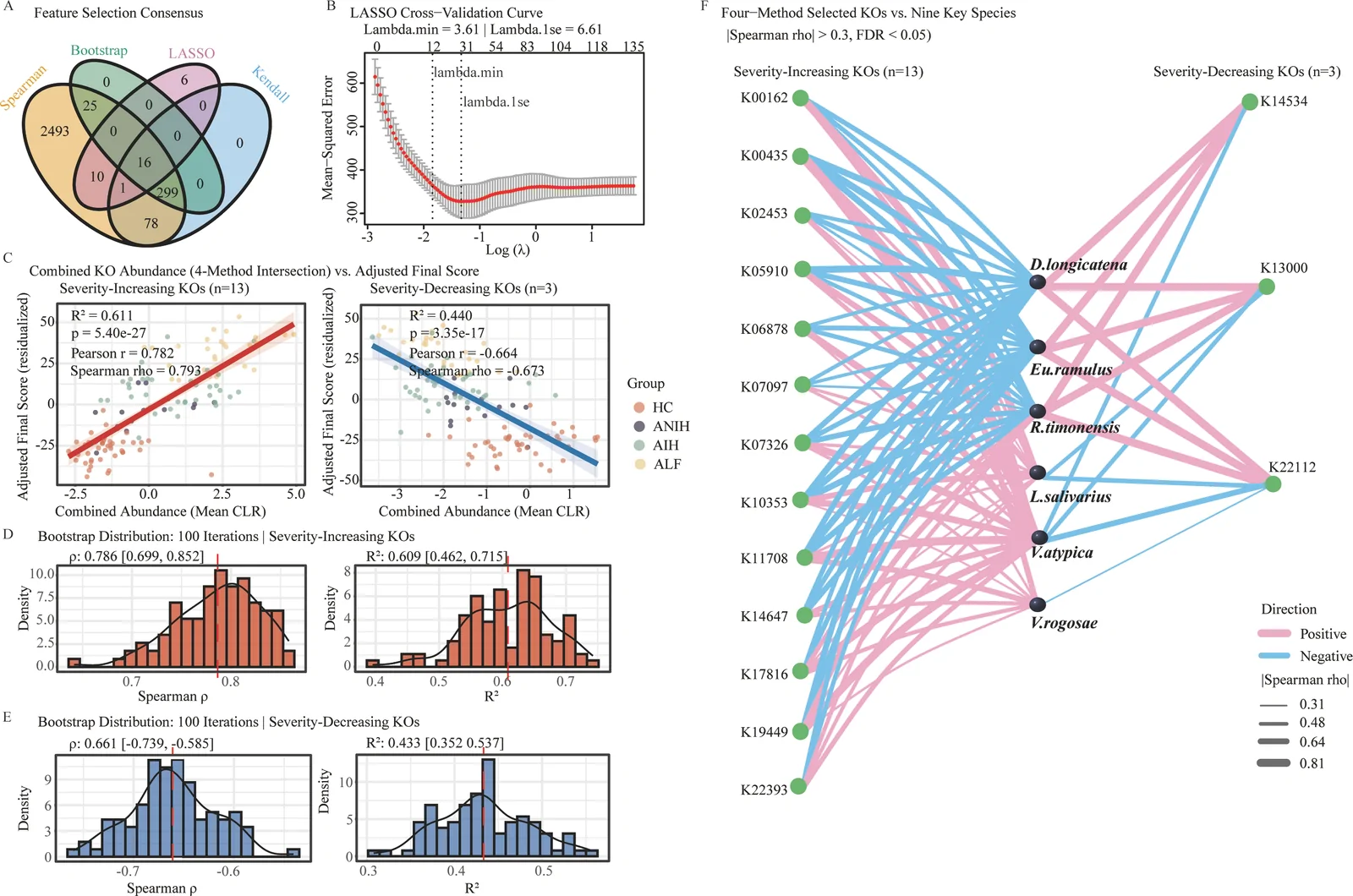
Functional gene selection, validation, and species-KO association network for four-method intersection KOs. (**A**) Venn diagram of the KO feature selection overlap across four methods: Spearman (|rho| > 0.3, FDR < 0.1), Kendall (|tau| > 0.3, FDR < 0.1), bootstrap (95% CI outside ± 0.3), and LASSO (lambda.min). (**B**) LASSO 10-fold cross-validation curve. Red dotted line: lambda.min = 3.785; black dashed line: lambda.1se = 6.313. Gray shading: ±1 SE across folds. (**C**) Scatter plots of combined CLR abundance versus residualized adjusted final score. Left panel, increasing KOs (Spearman rho > 0); right panel, decreasing KOs (Spearman rho < 0). Points colored by group; black line = linear fit with 95% CI. (**D–E**) Bootstrap distributions (*n* = 100, with replacement) of Spearman ρ (left) and *R*² (right) for the combined abundance (mean CLR) of four-method intersection KOs versus the residualized final score, shown separately for severity-increasing (**D**) and -decreasing KOs (**E**). Red dashed lines, bootstrap mean; gray dotted lines, 95% CI. (**G**) Network of significant KO-species correlations (|Spearman rho| ≥ 0.3, FDR < 0.05). HC, healthy controls; ANIH, acute non-icteric hepatitis; AIH, acute icteric hepatitis; ALF, acute liver failure.

Feature selection across four independent methods identified 16 core KOs at the stringent intersection, which expanded to 316 KOs when relaxing the threshold to at least three methods (Fig. 4A; Table S5a). LASSO cross-validation yielded lambda.min at 3.61 (33 KOs) and lambda.1se at 6.61 (12 KOs) (Fig. 4B). Stratification of the four-method intersection by correlation direction revealed 13 increasing KOs positively associated with disease severity (*R* squared = 0.611, *P* < 0.001, Spearman rho = 0.793) and three decreasing KOs showing the opposite trend (*R*² = 0.440, *P* < 0.001, Spearman rho = −0.673) both validated by bootstrap resampling (Fig. 4C and E; Table S5b). Network analysis linked increasing KOs negatively to butyrate producers (*R.timonensis*, *Eu. ramulus*, and *D. longicatena*) and positively to orally derived lactate utilizers (*V. rogosae*, *V. atypica*, and *L. salivarius*), with decreasing KOs showing the reverse pattern (Fig. 4F; Table S5c).

The less stringent at-least-three-method intersection identified 217 increasing and 99 decreasing KOs (Fig. S4A), with bootstrap validation confirming their significance (Fig. S4B and C; Table S5d). KEGG pathway enrichment of these 316 KOs revealed that score-positive pathways, including terpenoid backbone biosynthesis, ABC transporters, peptidoglycan biosynthesis, nitrogen metabolism, pyruvate metabolism, and two-component system, were negatively associated with butyrate producers and positively associated with orally derived lactate utilizers, whereas the score-negative pathways (glycolysis/gluconeogenesis, butanoate metabolism, and nitrotoluene degradation) showed the inverse taxonomic pattern (Fig. S4D and E; Table S5e). Group-wise pathway abundance comparisons supported these findings (Fig. S4F).

K08303 (lysine 2,3-aminomutase) abundance was significantly higher in HC than in all disease groups (Kruskal–Wallis with Dunn *post hoc* test, FDR < 0.01; Fig. S5A and B) and showed a modest negative correlation with clinical severity (Spearman rho = −0.346, *P* = 8.6 × 10⁻⁵; Fig. S5C). Its universal detection across all groups indicated that disease-associated variation is driven by quantitative abundance rather than presence–absence dynamics.

## DISCUSSION

Integration of XGBoost taxonomic modeling with multi-method functional selection revealed a dual-layered microbial signature in HEV severity. Taxonomic depletion of butyrate producers and enrichment of orally derived lactate utilizers was mirrored by coherent KO-level functional shifts against beneficial taxa and toward lactate-utilizing species, suggesting coupled ecological decay rather than independent drift. KEGG pathway enrichment implicated carbohydrate fermentation, nitrogen metabolism, and bacterial two-component signaling in this reprogramming. These patterns point to progressive ecological rewiring across the disease spectrum, though all inferences remain hypothesis generating and require functional validation.

This ecological rewiring was characterized by progressive enrichment of orally derived *Veillonella* species from HC to ALF, consistent with opportunistic colonization following barrier compromise. Their co-enrichment with *L. salivarius* implies a lactate-mediated ecological loop, a conserved motif in mucosal inflammatory conditions (27). Notably, *Streptococcus* species initially prioritized as lactate producers failed stricter significance testing, positioning the lactate axis as driven by *Veillonella* consumption and *L. salivarius* production rather than by *Streptococcus* overflow. This *Veillonella* enrichment has been reported in liver diseases (28, 29) and may promote barrier impairment, portal LPS influx, and hepatic inflammatory signaling, thereby contributing to disease progression (30). Reciprocally, butyrate producers, including *Roseburia* sp. MSJ-14 (31), *D. longicatena* (32), and *Eu. ramulus* (33), were progressively depleted, paralleling a decline in butanoate metabolism. This mirrors reports of the butyrate producers loss across chronic liver diseases, where reduced butyrate output compromises barrier integrity (34) and facilitates LPS translocation (35). Together, these opposing shifts suggest that HEV severity is associated with a coupled decay in which lactate-driven acidification displaces butyrate-mediated barrier maintenance.

The ecological shift was paralleled by directed functional remodeling at the KO level (36). While the 316-KO set captured attenuated signals, the 16 core KOs explained more variance, with increasing KOs enriched in pyruvate metabolism and lysine degradation and decreasing KOs in butanoate metabolism (K14534) and peptidoglycan biosynthesis (K22112 and K13000). This suggests HEV severity remodels microbial metabolism toward energy generation and stress response at the expense of cell wall maintenance. Notably, K14534, K22112, and K13000 were strongly negatively correlated with *V. atypica*, that *Veillonella*-driven lactate utilization may shift the metabolic balance away from butyrate production toward propionate formation (37) while suppressing peptidoglycan biosynthesis. Conversely, K08303 showed disease-associated but not severity-graded depletion. These inferences remain tentative, as metagenomics captures DNA abundance rather than transcriptional activity, requiring metabolomic validation.

Microbiome data provided only marginal incremental predictive value over clinical models, as biochemical indicators already accounted for the majority of variance in disease severity. Thus, microbiome signatures may serve as a complement to, rather than a replacement for, clinical scoring—potentially offering utility in early risk stratification or yielding mechanistic insights. Although the top taxa exhibited severity-associated signals independent of confounders, several limitations preclude definitive conclusions. The restricted age range (45–70 years) limits generalizability, and the cross-sectional design precludes causal inference. The modest size of the ANIH subgroup (*n* = 15) and the single-center design underscore the necessity for validation in larger, multi-center cohorts. Functional inferences drawn from metagenomic genetic potential await confirmation through targeted metabolomics or meta-transcriptomics. Furthermore, detailed information on medication uses and dietary intake was not available, although heavy drinkers and recent antibiotic users were excluded, and samples were collected prior to any interventions. Ultimately, independent replication in multi-center settings remains essential.

### Conclusion

This study provides a comprehensive, confounder-adjusted framework linking gut microbiome composition and functional capacity to HEV severity. Integration of machine-learning taxonomic modeling with multi-method feature selection identified *V. atypica* as a candidate severity-associated marker and delineated functional pathways suggestive of metabolic reprogramming across the disease spectrum. These findings offer correlational insights into gut–liver crosstalk and lay a preliminary foundation for hypothesis-driven research, pending external validation and functional confirmation.

## Supplementary Material

Reviewer comments

## Data Availability

Raw shotgun metagenomic sequencing data have been deposited in the NCBI BioProject database under accession numbers PRJNA1188794 and PRJNA1449747. Custom analysis scripts, including R code for XGBoost modeling, multi-method feature selection, and visualization, are available at https://github.com/Neal050617/Metagenomic_analysis_severe_Hepatitis_E_progression.

## SUPPLEMENTAL MATERIAL

Table S1a-c msystems.00956-26-s0001.xlsx Summary of subject-level clinical data and relative abundances of key bacterial species profiles.

Supplemental Checklist msystems.00956-26-s0002.docx STORMS checklist.

Supplemental File msystems.00956-26-s0003.docx Questionnaire, informed consent, additional experimental details, and supplemental figures and tables.

Table S1d-e msystems.00956-26-s0004.xlsx Microbial functional gene (KEGG Ortholog) profiles.

Table S2 msystems.00956-26-s0005.xlsx Construction and evaluation of a surrogate clinical severity index (final score) from routine blood biochemical and hematological parameters.

Table S3 msystems.00956-26-s0006.xlsx Comprehensive XGBoost classification model evaluation for discriminating hepatitis E patients from healthy controls.

Table S4 msystems.00956-26-s0007.xlsx GBoost regression model performance, feature importance, and incremental predictive value analyses for the continuous clinical severity score.

Table S5 msystems.00956-26-s0008.xlsx Bootstrap validation and functional gene (KO) association statistics for microbiome regression, combined abundance modeling, and species-gene correlation analyses.

OPEN PEER REVIEW reviewer-comments.pdf An accounting of the reviewer comments and feedback.

ASM does not own the copyrights to Supplemental Material that may be linked to, or accessed through, an article. The authors have granted ASM a non-exclusive, world-wide license to publish the Supplemental Material files. Please contact the corresponding author directly for reuse.
