# Supplementary material for: Clinical severity score-guided metagenomic analysis identifying gut microbial taxonomic and functional markers for severe hepatitis E progression
Source: mSystems. 2026 Sep 25;11(10):e00956-26. doi: 10.1128/msystems.00956-26 (PMC13712948; doi:10.1128/msystems.00956-26)
Supplement: Supplementary file 2 [file msystems.00956-26-s0002.docx]

Supplementary Table 1 - The STORMS checklist. An editable version for adaptation and inclusion in publications is available from <https://stormsmicrobiome.org>

| Number | Item | Recommendation | Item Source | Additional Guidance | Yes/No/NA | Comments or location in manuscript |
| --- | --- | --- | --- | --- | --- | --- |
| **Abstract** | | | | | | |
| 1.0 | Structured or Unstructured Abstract | Abstract should include information on background, methods, results, and conclusions in structured or unstructured format. | STORMS |  | Yes | Page 2 |
| 1.1 | Study Design | State study design in abstract. | STORMS | See 3.0 for additional information on study design. | Yes | Page 2 |
| 1.2 | Sequencing methods | State the strategy used for metagenomic classification. | STORMS | For example, targeted 16S by qPCR or sequencing, shotgun metagenomics, metatranscriptomics, etc. | Yes | Page 2 |
| 1.3 | Specimens | Describe body site(s) studied. | STORMS |  | Yes | Page 2 |
| **Introduction** | | | | | | |
| 2.0 | Background and Rationale | Summarize the underlying background, scientific evidence, or theory driving the current hypothesis as well as the study objectives. | STORMS |  | Yes | Page 4 |
| 2.1 | Hypotheses | State the pre-specified hypothesis. If the study is exploratory, state any pre-specified study objectives. | STORMS |  | Yes | Page 5 |
| **Methods** | | | | | | |
| 3.0 | Study Design | Describe the study design. | STORMS | Observational (Case-Control, Cohort, Cross-sectional survey, etc.) or Experimental (Randomized controlled trial, Non-randomized controlled trial, etc.). For a brief description of common study designs see: DOI: 10.11613/BM.2014.022  If applicable, describe any blinding (e.g. single or double-blinding) used in the course of the study. | NA | Blinding was not applicable in this cross-sectional study, as there was no intervention or group allocation. All laboratory analyses (DNA extraction, sequencing, and bioinformatics processing) were performed using standardized automated protocols, minimizing the potential for subjective bias. |
| 3.1 | Participants | State what the population of interest is, and the method by which participants are sampled from that population. Include relevant information on physiological state of the subjects or stage in the life history of disease under study when participants were sampled. | STORMS | Examples of the population of interest could be: adults with no chronic health conditions, adults with type II diabetes, newborns, etc. This is the total population to whom the study is hoped to be generalizable to. The sampling method describes how potential participants were selected from that population.  If the participants are from a substudy of a larger study, provide a brief description of that study and cite that study.  Clearly state how cases and controls are defined.  An example of relevant physiological state might be pre/post menopausal for a vaginal microbiome study; examples of stage in the life history of disease could be whether specimens were collected during active or dormant disease, or before or after treatment. | Yes | Page 6 |
| 3.2 | Geographic location | State the geographic region(s) where participants were sampled from. | MIxS: geographic location (country and/or sea,region) | Geographic coordinates can be reported to prevent potential ambiguities if necessary. | Yes | line 16-17 in Page 6 |
| 3.3 | Relevant Dates | State the start and end dates for recruitment, follow-up, and data collection. | STORMS | Recruitment is the period in which participants are recruited for the study. In longitudinal studies, follow-up is the date range in which participants are asked to complete a specific assessment. Finally, data collection is the total period in which data is being collected from participants including during initial recruitment through all follow-ups. | NA | Study Design: Observational (Cross-sectional)  Blinding: NA  Relevant Dates:  Recruitment: December 2022 to May 2025 (Line 11 in Page 6)    Follow-up: NA |
| 3.4 | Eligibility criteria | List any criteria for inclusion and exclusion of recruited participants. | Modified STROBE | Among potential recruited participants, how were some chosen and others not? This could include criteria such as sex, diet, age, health status, or BMI.  If there is a primary and validation sample, describe inclusion/exclusion criteria for each. | Yes | Line 11-20 in Page 6 |
| 3.5 | Antibiotics Usage | List what is known about antibiotics usage before or during sample collection. | STORMS | If participants were excluded due to current or recent antibiotics usage, state this here.  Other factors (e.g. proton pump inhibitors, probiotics, etc.) that may influence the microbiome should also be described as well. | Yes | Page 6 and 7; “participants were excluded due to current or recent antibiotics usage” |
| 3.6 | Analytic sample size | Explain how the final analytic sample size was calculated, including the number of cases and controls if relevant, and reasons for dropout at each stage of the study. This should include the number of individuals in whom microbiome sequencing was attempted and the number in whom microbiome sequencing was successful. | STORMS | Consider use of a flow diagram (see template at https://stormsmicrobiome.org/figures). Also state sample size in abstract.  If power analysis was used to calculate sample size, describe those calculations. | Yes | Line 1-11 in Page 7 |
| 3.7 | Longitudinal Studies | For longitudinal studies, state how many follow-ups were conducted, describe sample size at follow-up by group or condition, and discuss any loss to follow-up. | STORMS | If there is loss to follow-up, discuss the likelihood that drop-out is associated with exposures, treatments, or outcomes of interest. | NA |  |
| 3.8 | Matching | For matched studies, give matching criteria. | Modified STROBE | "Matched" refers to matching between comparable study participants as cases and controls or exposed / unexposed.  Indicate whether participants were individual or frequency matched and in what ratio were they matched (e.g. 1 case to 1 control). | Yes | Line 11 in Page 7 |
| 3.9 | Ethics | State the name of the institutional review board that approved the study and protocols, protocol number and date of approval, and procedures for obtaining informed consent from participants. | STORMS |  | Yes | Lines 7-8 in Page 6 |
| 4.0 | Laboratory methods | State the laboratory/center where laboratory work was done. | STORMS | Provide a reference to complete lab protocols if previously published elsewhere such as on protocols.io. Note any modifications of lab protocols and the reason for protocol modifications. | Yes | Line 1 in Page 8 for blood samples; and Line 4 in Page 8 for fecal samples. |
| 4.1 | Specimen collection | State the body site(s) sampled from and how specimens were collected. | MIxS: sample collection device or method; host body site | Use terms from the Uber-anatomy Ontology (https://www.ebi.ac.uk/ols/ontologies/uberon) to describe body sites in a standardized format. | Yes | Line 1 in Page 8:  blood samples; and Line 4 in Page 8: fecal samples. |
| 4.2 | Shipping | Describe how samples were stored and shipped to the laboratory. | STORMS | Include length of time from collection to receipt by the lab and if temperature control was used during shipping. | NA | samples were collected and processed within the same hospital facility; no shipping was required |
| 4.3 | Storage | Describe how the laboratory stored samples, including time between collection and storage and any preservation buffers or refrigeration used. | STORMS | State where each procedure or lot of samples was done if not all in the same place.  Include reagent/lot/catalogue #s for storage buffers. | Yes | Line 22 in page 7 |
| 4.4 | DNA extraction | Provide DNA extraction method, including kit and version if relevant. | MIxS: nucleic acid extraction | If any DNA quantification methods were used prior to DNA amplification or at the pooling step of library preparation, state so here. | Yes | Lines 3-4 in Page 8 |
| 4.5 | Human DNA sequence depletion or microbial DNA enrichment | Describe whether human DNA sequence depletion or enrichment of microbial or viral DNA was performed. | STORMS |  | NA | Human sequences were removed during bioinformatics preprocessing by aligning reads to the human reference genome and discarding mapped reads (in silico depletion |
| 4.6 | Primer selection | Provide primer selection and DNA amplification methods as well as variable region sequenced (if applicable). | MIxS: pcr primers |  | NA | shotgun metagenomics; no PCR amplification was performed |
| 4.7 | Positive Controls | Describe any positive controls (mock communities) if used. | STORMS | If used, should be deposited under guidance provided in the 8.X items. | Yes | Custom mock community composed of laboratory‑cultured *Bifidobacterium longum* and *Escherichia coli* was used as a positive control. Bacterial concentrations were determined by plate dilution colony counting and optical density (OD) measurement.  ines17-21 in Page 8, and details were shown Page 7 in Supplementary file |
| 4.8 | Negative Controls | Describe any negative controls if used. | STORMS | If used, should be deposited under guidance provided in the 8.X items. | Yes | Sterile, nuclease‑free water was used as a negative control in each batch of DNA extraction and library preparation. Lines17-21 in Page 8, and details were shown Page 7 in Supplementary file; |
| 4.9 | Contaminant mitigation and identification | Provide any laboratory or computational methods used to control for or identify microbiome contamination from the environment, reagents, or laboratory. | STORMS | Includes filtering of reagents and other steps to minimize contamination. It is relevant to state whether the specimens of interest have low microbial load, which makes contamination especially relevant. | Yes | Both laboratory and computational methods were applied to control and identify potential contamination. Details are shown in Page 7 in Supplementary file. |
| 4.10 | Replication | Describe any biological or technical replicates included in the sequencing, including which steps were replicated between them. | STORMS | Replication may be biological (redundant biological specimens) or technical (aliquots taken at different stages of analysis) and used in extraction, sequencing, preprocessing, and/or data analysis. | Biological replicates were included in the study design. No technical replicates were performed. | Biological replicates were included in the study design, with multiple independent participants within each study group (HE patients and healthy controls). Each participant represents an independent biological replicate. No technical replicates were performed for any sample.Biological replication provides sufficient statistical power for group‑level comparisons. Details are shown in Page 7 in Supplementary file. |
| 4.11 | Sequencing strategy | Major divisions of strategy, such as shotgun or amplicon sequencing. | MIxS: sequencing method | For amplicon sequencing (for example, 16S variable region), state the region selected. State the model of sequencer used. | Yes | Line 15 in Page 8 |
| 4.12 | Sequencing methods | State whether experimental quantification was used (QMP/cell count based, spike-in based) or whether relative abundance methods were applied. | STORMS | These include read length, sequencing depth per sample (average and minimum), whether reads are paired, and other parameters. | Yes | Line 14-15 in Page 8 |
| 4.13 | Batch effects | Detail any blocking or randomization used in study design to avoid confounding of batches with exposures or outcomes. Discuss any likely sources of batch effects, if known. | STORMS | Sources of batch effects include sample collection, storage, library preparation, and sequencing and are commonly unavoidable in all but the smallest of studies. | Yes | Samples were processed in three sequencing batches. Batch effects were corrected computationally using MMUPHin (adjust_batch function), with sequencing batch included as a covariate in the correction model. More details were shown in Supplementary file. |
| 4.14 | Metatranscriptomics | Detail whether any mRNA enrichment was performed and whether/how retrotranscription was performed prior to sequencing. Provide size range of isolated transcripts. Describe whether the sequencing library was stranded or not. Provide details on sequencing methods and platforms. | STORMS | Provide details on any internal standards which may have been used as well as parameters and versions of any software or databases used. | NA |  |
| 4.15 | Metaproteomics | Detail which protease was used for digestion. Provide details on proteomic methods and platforms (e.g. LC-MS/MS, instrument type, column type, mass range, resolution, scan speed, maximum injection time, isolation window, normalised collision energy, and resolution). | STORMS | Provide details on any internal standards which may have been used as well as parameters and versions of any software or databases used. | NA |  |
| 4.16 | Metabolomics | Specify the analytic method used (such as nuclear magnetic resonance spectroscopy or mass spectrometry). For mass spectrometry, detail which fractions were obtained (polar and/or non-polar) and how these were analyzed. Provide details on metabolomics methods and platforms (e.g. derivatization, instrument type, injection type, column type and instrument settings). | STORMS | Provide details on any internal standards which may have been used as well as parameters and versions of any software or databases used. | NA |  |
| 5.0 | Data sources/  measurement | For each non-microbiome variable, including the health condition, intervention, or other variable of interest, state how it was defined, how it was measured or collected, and any transformations applied to the variable prior to analysis. | MIxS: host disease status | State any sources of potential bias in measurements, for example multiple interviewers or measurement instruments, and whether these potential biases were assessed or accounted for in study design.  Use terms from a standardized ontology such as the Experimental Factor Ontology (https://www.ebi.ac.uk/efo/) to describe variables of interest in a standardized format. | Yes | All non-microbiome variables were collected via structured questionnaires and medical history reviews at enrollment. Variables included demographic information, lifestyle factors, and clinical characteristics. Dietary assessment: Participants reported typical daily meals characteristic of the Zhejiang region (rice-based with vegetables and meat).Lines 13-17 in Page 7. |
| 6.0 | Research design for causal inference | Discuss any potential for confounding by variables that may influence both the outcome and exposure of interest. State any variables controlled for and the rationale for controlling for them. | STORMS | For causal inference, this item refers to describing the assumptions that would be required to draw causal inferences from observational data. See Vujkovic-Cvijin, I., Sklar, J., Jiang, L. et al. Host variables confound gut microbiota studies of human disease. Nature 587, 448–454 (2020). https://doi.org/10.1038/s41586-020-2881-9 for more details on confounding in observational microbiome studies.  For example, hypothesized confounders may be controlled for by multivariable adjustment. Consider using a directed acyclic graph (DAG) to describe your causal model and justify any variables controlled for. DAGs can be made using [www.dagitty.net](http://www.dagitty.net/). | Yes | We controlled for potential confounding by age, sex, and BMI through multivariable adjustment in all regression models. These variables were selected a priori based on established literature demonstrating their association with both gut microbiome composition and disease severity in hepatitis E. Given the observational nature of our cross‑sectional study, causal inference cannot be established; our findings should be interpreted as associational. |
| 6.1 | Selection bias | Discuss potential for selection or survival bias. | STORMS | Selection bias can occur when some members of the target study population are more likely to be included in the study/final analytic sample than others. Some examples include survival bias (where part of the target study population is more likely to die before they can be studied), convenience sampling (where members of the target study population are not selected at random), and loss to follow-up (when probability of dropping out is related to one of the things being studied). | NA | Potential selection bias was minimized by recruiting participants from multiple sources and applying consistent inclusion/exclusion criteria across all groups. However, convenience sampling from a single geographic region (Zhejiang, China) may limit generalizability. No survival bias or loss‑to‑follow‑up is applicable due to the cross‑sectional design.  Detail was shown in Method section. For example, Line 11-12 in Page 10; Line 8-10 in Page 11; Line 1-12 in Page 12; Line 21 in Page 13 to Line 7 in Page 14, etc. |
| 7.0 | Bioinformatic and Statistical Methods | Describe any transformations to quantitative variables used in analyses (e.g. use of percentages instead of counts, normalization, rarefaction, categorization). | STORMS | If a variable is analyzed using different transformations, state rationale for the transformation and for each analyses which version of the variable is used.  In case of any complex or multistep transformations, give enumerated instructions for reproducing those transformations. | Yes | Multiple transformations were applied to different data types. Species and functional gene abundances were CLR‑transformed to address compositionality. Clinical variables were standardized before model fitting. All transformations are described in detail in Method section. For example, Line 15 in Page 11; Line 14 in Page 14; Supplementary Table S2d, etc. |
| 7.1 | Quality Control | Describe any methods to identify or filter low quality reads or samples. | MIxS: sequence quality check | If samples were excluded based on quality or read depth, list the criteria used, the number of samples excluded, and the final sample size after quality control. | Yes | Raw paired-end reads were first assessed using FastQC and summarized with MultiQC. Quality filtering and adapter trimming were performed using Trimmomatic within KneadData, with ILLUMINACLIP adapter removal, a 4-bp sliding window Q-score cutoff of 20, and a minimum post-trimming read length of 50 bp. Reads containing ambiguous bases and host-derived reads were removed during quality control and host-decontamination steps. No samples were excluded based on sequencing depth. |
| 7.2 | Sequence analysis | Describe any taxonomic, functional profiling, or other sequence analysis performed. | MIxS: feature prediction; similarity search method |  | Yes | Line 10 in Page 9 to Line 5 in Page 10 |
| 7.3 | Statistical methods | Describe all statistical methods. | Modified STROBE | Describe any statistical tests used, exploratory data analysis performed, dimension reduction methods/unsupervised analysis, alpha/beta metrics, and/or methods for adjusting for measurement bias.  If multiple statistical methods are possible, discuss why the methods used were selected.  If a multiple hypothesis testing correction method was used, describe the type of correction used.  State which taxonomic levels are analyzed. | Yes | Lines 9-14 in Page 16 for bacteria species abundance and prevalence; Lines 5-10 in Page 18 for bacterial genes abundance. |
| 7.4 | Longitudinal analysis | If the study is longitudinal, include a section that explicitly states what analysis methods were used (if any) to account for grouping of measurements by individual or patterns over time. | STORMS |  | NA | cross‑sectional study; no longitudinal follow‑up or repeated measurements were performed |
| 7.5 | Subgroup analysis | Describe any methods used to examine subgroups and interactions. | STROBE |  | NA | No subgroup analyses were performed. Age, sex, and BMI were controlled as confounders but were not tested as effect modifiers. |
| 7.6 | Missing data | Explain how missing data were addressed. | STROBE | "Missing data" refers to participant measurements such as covariates, exposures, outcomes, or time points that should have been collected but were not, not to zeros in taxonomic abundance tables or data points not applicable to that observation. | No |  |
| 7.7 | Sensitivity analyses | Describe any sensitivity analyses. | STROBE |  | Yes | Line 21 in Page 12 to Line 7 in Page 13; Lines 16-21 in Page 17 |
| 7.8 | Findings | State criteria used to select findings for reporting. | STORMS | For example, false discovery rate with total number of tests, effect size threshold, significance threshold, microbes of interest. | Yes | A multi‑step selection strategy was used to identify core microbial taxa associated with disease severity: (1) feature ranking by a composite score integrating XGBoost Gain, monotonicity, and Spearman correlation; (2) stability assessment via 100 bootstrap iterations and 5‑fold CV; and (3) permutation testing (100×) to confirm significance. Details were shown in Method section, such as Lines 7-20 in Page 13; Line 21 in Page 14 to Line 8 in Page 15. |
| 7.9 | Software | Cite all software (including read mapping software) and databases (including any used for taxonomic reference or annotating amplicons, if applicable) used. Include version numbers. | Modified STREGA | Installed packages, add-ons or libraries should be stated and cited in addition to the software used.  All parameters employed that differ from the default of that software/version should be provided.  This is in addition to, not a replacement for, publishing of code as outlined in the section Reproducible Research. | Yes | "xgboost", "caret", "ggplot2", "openxlsx", "ggrepel",  "patchwork", "RColorBrewer", "viridis", "tidyr", "dplyr",  "ggdist", "MASS", "broom", "psych"  “pROC”  “ggplot2”  “glmnet”, etc, Detail were shown in the R scripts: <https://github.com/Neal050617/Metagenomic_analysis_severe_Hepatitie_E_progression>. |
| 8.0 | Reproducible research | Make a statement about whether and how others can reproduce the reported analysis. | STORMS | Any protected information that has been excluded or provided under controlled access should be listed along with any relevant data access procedures. "On request from authors" is not sufficiently detailed; formal data access procedures and conditions should be defined.  If data are unavailable, state so clearly.  Consider using a specialized rubric for reproducible research (such as: <https://mbio.asm.org/content/9/3/e00525-18.short)>.  Consider preregistering the study protocol (such as o[n osf.](http://osf.io/)io or <https://plos.org/open-science/preregistration/).> | Yes | Raw sequencing data are available at NCBI BioProject (PRJNA1188794 and PRJNA1449747). Analysis code (XGBoost modeling, feature selection, visualization) is available at <https://github.com/Neal050617/Metagenomic_analysis_severe_Hepatitie_E_progression> and has been repeatedly executed to verify result reproducibility. Participant clinical data are not publicly available due to privacy restrictions but may be accessed via the corresponding author under institutional data access policies. |
| 8.1 | Raw data access | State where raw data may be accessed including demultiplexing information. | STORMS | Robust, long-term databases such as those hosted by NCBI and EBI are preferred. If using a private repository, provide rationale. | Yes | Raw shotgun metagenomic sequencing data have been deposited in the NCBI BioProject database under accession numbers **PRJNA1188794** and **PRJNA1449747**. And Supplementary Table S1. |
| 8.2 | Processed data access | State where processed data may be accessed. | STORMS | Unfiltered data should be provided.  Robust, long-term databases such as those hosted by NCBI and EBI-EMBL are preferred. Repositories like zenodo (https://zenodo.org/) or publisso (https://www.publisso.de/en/working-for-you/doi-service/)  can be used to provide a DOI and long-term storage for processed datasets, even those which cannot be published openly. | Yes | Processed data, including species abundance tables, functional gene (KO) abundance matrices, and core feature selection results, are available at Supplementary Table S1. |
| 8.3 | Participant data access | State where individual participant data such as demographics and other covariates may be accessed, and how they can be matched to the microbiome data. | STORMS | If re-categorized, transformed, or otherwise derived variables were used in the analysis, these variables or code for deriving them should be provided.  Examples of how participant data can be matched to microbiome data are: using the same set of anonymized identifiers, or using different anonymized identifiers but providing a map.  Provided data should be sufficient to independently replicate the current analysis. | Yes | De‑identified participant data, including demographics (age, sex, BMI), clinical characteristics, and lifestyle factors, are not publicly available due to privacy restrictions. These data are available from the corresponding author upon reasonable request, subject to institutional data access policies and ethics committee approval. Participant data and microbiome data can be matched using the same anonymized sample IDs. Details were shown in Supplementary Table S1 |
| 8.4 | Source code access | State where code may be accessed. | STORMS | If a standard or formalized workflow was employed, reference it here. | Yes | Detail were shown in the R scripts: <https://github.com/Neal050617/Metagenomic_analysis_severe_Hepatitie_E_progression>. |
| 8.5 | Full results | Provide full results of all analyses, in computer-readable format, in supplementary materials. | STORMS | For example, any fold-changes, p-values, or FDR values calculated, provided as a spreadsheet.  Use a machine-readable, plain-text format such as csv or tsv. | Yes | Details were shown in Supplementary Table S2,S3, S4 and S5. |
| **Results** | | | | | | |
| 9.0 | Descriptive data | Give characteristics of study participants (e.g. dietary, demographic, clinical, social) and information on exposures and potential confounders. | STROBE | Typically reported in a table included in the paper or as a supplementary table. Indicate number of participants with missing data for each variable of interest.  This includes environmental and lifestyle factors that may affect the relationship between the microbiome and the condition of interest. Participant diet and medication use should be summarized, if known.  At minimum, age and sex of all participants should be summarized. | Yes | Participant characteristics, including age, sex, BMI, disease severity groups, lifestyle factors, and clinical parameters, are shown in Supplementary Table S1. No missing data for key variables. |
| 10.0 | Microbiome data | Report descriptive findings for microbiome analyses with all applicable outcomes and covariates. | STORMS | This includes measures of diversity as well as relative abundances. These descriptive findings should be reported both for the sample overall and for individual groups. | Yes | Descriptive microbiome findings, including alpha diversity, beta diversity, and taxonomic/functional composition, are reported for the overall cohort and by disease severity groups. Details were shown in Supplementary FigS1, and Supplementary Table S1c and S1e; Lines 13-22 in Page 18 |
| 10.1 | Taxonomy | Identify taxonomy using standardized taxon classifications that are sufficient to uniquely identify taxa. | STORMS | If not using full taxonomic hierarchy, make sure it is clear whether names stated are species, genera, family, etc.  Italicize genus/species pairs. Consult journal guidelines or standardized references on taxonomic nomenclature. For instance, <https://wwwnc.cdc.gov/eid/page/scientific-nomenclature> | Yes | axonomy was assigned using a custom database of universal single-copy genes (USCGs) derived from OrthoDB. Clean reads were aligned to this USCG database using minimap2, and taxonomic abundances were calculated as reads per kilobase per million (RPKM) for each taxon per sample. Detail were shown in Supplementary S1b |
| 10.2 | Differential abundance | Report results of differential abundance analysis by the variable of interest and (if applicable) by time, clearly indicating the direction of change and total number of taxa tested. | STORMS | If there are more than two groups, include omnibus (multigroup) test results if applicable to the research question.  If applicable, reported effect sizes should include a measure of uncertainty such as the confidence interval. | Yes | Details were shown in Supplementary FigS2 and S3F. |
| 10.3 | Other data types | Report other data analyzed--e.g. metabolic function, functional potential, MAG assembly, and RNAseq. | STORMS |  | Yes | Metabolic functional potential was assessed via shotgun metagenomic sequencing. KO abundance profiles are reported in Supplementary Table S2d and S5e. |
| 10.4 | Other statistical analysis | Report any statistical data analysis not covered above. | STORMS | This could include subgroup analysis, sensitivity analyses, and cluster analysis.  Visualizations should be easily interpretable and colorblind-friendly. The caption and/or main text should provide a detailed description of visualizations for visually-impaired readers. | Yes | sensitivity analyses were detailed in Method setion. Lines1-21 in Page 21 |
| **Discussion** | | | | | | |
| 11.0 | Key results | Summarise key results with reference to study objectives | STROBE |  |  |  |
| 12.0 | Interpretation | Give a cautious overall interpretation of results considering objectives, limitations, multiplicity of analyses, results from similar studies, and other relevant evidence. | STROBE | Define or clarify any subjective terms such as "dominant," "dysbiosis," and similar words used in interpretation of results.  When interpreting the findings, consider how the interpretation of the findings may be summarized or quoted for the general public such as in press releases or news articles.  If causal language is used in the interpretation (such as "alters," "affects," "results in," "causes," or "impacts"), assumptions made for causal inference should be explicitly stated as part of 6.0 and 13.0.  Distinguish between function potential (ie inferred from metagenomics) and observed activity (ie metatranscriptomic, metabolomic, proteomic) if discussing microbial function. | Yes | Our findings demonstrate that gut microbial taxonomic and functional shifts—particularly lactate‑utilizing Veillonella enrichment and beneficial commensal depletion—are consistently associated with HE severity. These associations are correlational, not causal, and should be interpreted as hypothesis‑generating. Functional interpretations are based on metagenomic predictions (KEGG Orthologs) and require metabolomic or metatranscriptomic confirmation. The term “dysbiosis” is used to describe deviation from the healthy control community structure. Details were shown in Discussion setion. |
| 13.0 | Limitations | Discuss limitations of the study, taking into account sources of potential bias or imprecision. | STROBE | Also consider limitations resulting from the methods (especially novel methods), the study design, and the sample size. | Yes | Details was shown in Lines 4-16 in Page 18. |
| 13.1 | Bias | Discuss any potential for bias to influence study findings. | STORMS | May include sampling method, representativeness of study participants, or potential confounding. | Yes | Details was shown in Lines 3-4 in Page 26. |
| 13.2 | Generalizability | Discuss the generalisability (external validity) of the study results | STROBE | To what populations or other settings do you expect the conclusions to generalize? | Yes | Our findings are primarily generalizable to adult Chinese patients with acute hepatitis E in a single‑center setting. The results require validation in multi‑center, diverse cohorts. These findings offer correlational insights into gut–liver crosstalk and lay a preliminary foundation for hypothesis‑driven research, pending external validation and functional confirmation (see Lines 6-19 in Page 26) |
| 14.0 | Ongoing/future work | Describe potential future research or ongoing research based on the study's findings. | STORMS |  | Yes | Details were shown in Lines 6-19 in Page 26. Functional inferences drawn from metagenomic genetic potential await confirmation through targeted metabolomics or meta‑transcriptomics. |
| **Other information** | | | | | | |
| 15.0 | Funding | Give the source of funding and the role of the funders for the present study and, if applicable, for the original study on which the present article is based | STROBE |  | Yes | Lines 4-9 in Page 28 |
| 15.1 | Acknowledgements | Include acknowledgements of those who contributed to the research but did not meet critera for authorship. | STORMS | For general guidelines on authorship, see [http://www.icmje.org](http://www.icmje.org/) and <https://www.elsevier.com/authors/journal-authors/policies-and-ethics/credit-author-statement> | Yes | Lines 12-15 in Page 28 |
| 15.2 | Conflicts of Interest | Include a conflicts of interest statement. | STORMS |  | Yes | Line 17 in Page 28 |
| 16.0 | Supplements | Indicate where supplements may be accessed and what materials they contain. | STORMS |  | Yes | Page 38-47 |
| 17.0 | Supplementary data | Provide supplementary data files of results with for all taxa and all outcome variables analyzed. Indicate the taxonomic level of all taxa. | STORMS | Depending on the analysis performed, examples of the supplemental results included could be mean relative abundance, differential abundance, raw p-value, multiple hypothesis testing-adjusted p-values, and standard error.  All discussed taxa should include the taxonomic level (e.g. class, order, genus). | Yes | See Supplementary file |
