# Supplementary material for: Clinical severity score-guided metagenomic analysis identifying gut microbial taxonomic and functional markers for severe hepatitis E progression
Source: mSystems. 2026 Sep 25;11(10):e00956-26. doi: 10.1128/msystems.00956-26 (PMC13712948; doi:10.1128/msystems.00956-26)
Supplement: Supplementary file 3 [file msystems.00956-26-s0003.docx]

# Supplementary Files

**The attached catalogue:**

1. **Informed consent form (Translated from Chinese)	2**
2. **Informed consent form for scientific research (Translated from Chinese) 3**
3. **Questionnaire	5**
4. **Supplementary material and methods	7**
5. **Supplementary Fig S1-S5	8**
6. **Supplementary data S1-S5	14**

1. **Informed consent form (Translated from Chinese).**

We are from the Department of Thyroid Surgery and the State Key Laboratory for Diagnosis and Treatment of Infectious Diseases, the National Clinical Research Center for Infectious Diseases, the National Medical Center for Infectious Diseases, the Collaborative Innovation Center for Diagnosis and Treatment of Infectious Diseases, The First Affiliated Hospital, School of Medicine, Zhejiang University. We will collect your fecal and blood samples to analyze whether the exacerbation of viral hepatitis type E (HE) is always associated with intestinal microbiota dysbiosis and identify HE exacerbation-associated microbial indicators. These findings will contribute to the development of multifactorial therapeutic and preventive management strategies in the future. Please provide your fecal and blood samples according to our instructions. The entire process is free of charge, and these results will be used for scientific research only. Thank you for your cooperation.

Number：

Patient’s Signature：

Date:

2. **Informed consent form for scientific research.**

Dear participants,

We are from the State Key Laboratory for Diagnosis and Treatment of Infectious Diseases, the National Clinical Research Center for Infectious Diseases, the National Medical Center for Infectious Diseases, the Collaborative Innovation Center for Diagnosis and Treatment of Infectious Diseases, The First Affiliated Hospital, School of Medicine, Zhejiang University. We will gather information about your health condition and record your clinical data. The fecal and blood samples will be used for scientific research. The resulting data will help characterize intestinal microbiota dynamics in the progress of viral hepatitis type E (HE). Thank you for your cooperation.

You may choose not to participate in this study. If you decide to participate, please sign this informed consent form to indicate your agreement. Before starting the study, the researchers will screen you to determine if you are a suitable candidate. If you choose to participate, we hope you will commit to completing the entire study process.

The subjects of this study are individuals with HEV infection who do not have other major diseases. We will collect health status information from participants through questionnaires. We will collect fecal and blood samples twice: enrolment and one-year postdischarge follow-up visit. Fecal samples will be used to extracted microbial DNA which used for shotgun sequencing, and blood samples will undergo routine blood tests and biochemistry examinations.

There is no cost associated with participating in this study.

The information collected during the research will be kept strictly confidential and accessible only to researchers involved in the study. Your name will be replaced with numbers, and only the researchers will have access to the corresponding information. It will not be shared with or disclosed to anyone outside our research team.

The findings from this research may be published in a journal to share insights with other interested parties, but your confidential information will not be disclosed. You will have access to information and updates regarding this study’s process. If you have any questions about the study, please contact the researcher.

If you comprehend the content of the study and agree to participate, please sign this informed consent. A copy of this form will be given to you for your records.

**Statement by the participant**:

I confirm that I have read the informed consent form for this study or have had it read to me. I have had the opportunity to ask questions about this study, and any potential issues and their solutions have been explained to me. I understand that my participation in the study is voluntary and that choosing not to participate will not affect any of my entitled benefits.

I understand that the researchers involved in this study have the right to review the study records and case data. I consent to their direct access to my study records, knowing that this information will be handled confidentially.

I voluntarily consent to participate in this research.

(Participants who are illiterate should also include their thumbprint.)

Print Name of Participant__________________

Signature of Participant ___________________

Thumbprint of participant_________________

Date ___________________________

Day/month/year

A literate witness must sign; ideally, this person should be chosen by the participant and have no connection to the research team).

Print name of witness_____________________

Signature of witness _____________________

Date ________________________

Day/month/year

Statement by the researcher/person taking consent

I have accurately read out the information sheet to the potential participant and have ensured, to the best of my ability, that the participant understands that the following:

1. We will collect information about your health condition and record your clinical data.

2. These data will be used for scientific research.

3. The collected fecal and blood samples will be used for scientific research.

I confirm that the participant was given an opportunity to ask questions about the study, and all the questions were answered satisfactorily. I also confirm that the participant was not coerced into giving consent.

Print Name of Researcher/person taking the consent________________________

Signature of Researcher /person taking the consent__________________________

Date ___________________________

Day/month/year

3. **Questionnaire**

**Questionnaire**

**Questionnaire (Age>18 years)**

**Name：	Age：	Gender：**

**Height：	cm Weight：	kg**

**（1）What is your place of residence?**

**- [ ] urban**

**- [ ] rural**

（2）**If positive labled “√”, and negative labled “X”**

**- [ ] HAV-IgM, [ ] HAV-IgG**

**- [ ] HBV-IgM, [ ] HBV-IgG**

**- [ ] HCV-IgM, [ ] HCV-IgG**

**- [ ] HDV-IgM, [ ] HDV-IgG**

**- [ ] HEV-IgM, [ ] HEV-IgG**

（3）**Eating habits**

**- [ ] rice**

**- [ ] vegetable and meet**

**- [ ] noodles**

**- [ ] Yogurt and other dairy products**

**- [ ] Yogurt and other dairy products**

**- [ ] excessive alcohol**

**- [ ] excessive cigarette**

**- [ ] drug consumption**

（4）**Have you ever used antibiotics？**

**- [ ] never used antibiotics**

**- [ ] previous use of antibiotics, no antibiotic use within 1 month**

**- [ ] previous use of antibiotics, antibiotic use within 1 month**

**（9）Do you often stay up late?**

**- [ ] yes**

**- [ ] no**

**（10）Have you experienced any of the following digestive problems? (Multiple options available)**

**- [ ] frequent bloating**

**- [ ] frequent diarrhea**

**- [ ] frequent acid reflux**

**- [ ] frequent abdominal pain**

**- [ ] frequent constipation**

**- [ ] other digestive diseases, please specify the name of the disease_________**

**- [ ] no digestive diseases**

**（10）Have you experienced any of the following problems? (Multiple options available)**

**- [ ] solid organ transplant recipients**

**- [ ] infected with human immunodeficiency virus (HIV)**

**（11）Have you experienced any of the following liver problems? (Multiple options available)**

**- [ ]autoimmune hepatitis**

**- [ ]primary biliary cholangitis**

**- [ ]PSC**

**- [ ] hepatocellular carcinoma**

**- [ ] drug-induced liver injury**

4. **Supplementary material and methods**

**Quality Controls for DNA extraction, library preparation, and sequencing.**

A custom positive control comprising laboratory‑cultured *Bifidobacterium longum* and *Escherichia coli* was included in each sequencing batch to monitor DNA extraction efficiency and sequencing performance. Bacterial concentrations were determined by plate counting and OD₆₀₀ measurement. In parallel, sterile nuclease‑free water was processed as a negative control to detect potential reagent or environmental contamination. The positive control consistently yielded the expected microbial composition, while the negative control showed no significant microbial reads, confirming the reliability of the experimental workflow. Contamination was minimized at the laboratory level by using sterile, nuclease‑free reagents, filter‑tips, and dedicated PCR‑free clean areas for DNA extraction and library preparation. Negative controls (sterile water) were processed in parallel with study samples through the entire workflow to monitor reagent and environmental contamination. In addition, all extraction and library preparation steps were performed in a laminar flow hood with UV sterilization before each use.

Biological replication was achieved by including multiple independent participants in each study group (ANIH, n=15; AIH, n=38; ALF, n=28; healthy controls, n= 44). No technical replicates were performed for DNA extraction or sequencing. This approach is consistent with the study aim to compare microbial composition at the group level, where biological replication provides adequate statistical power to detect differences between groups, and is widely used in large‑scale microbiome studies.

Raw paired-end reads were first assessed using FastQC and summarized with MultiQC. Quality filtering and adapter trimming were performed using Trimmomatic within KneadData, with ILLUMINACLIP adapter removal, a 4-bp sliding window Q-score cutoff of 20, and a minimum post-trimming read length of 50 bp. Reads containing ambiguous bases and host-derived reads were removed during quality control and host-decontamination steps.

To account for potential batch effects arising from three independent sequencing runs, we applied MMUPHin (version 2.1.0; Meta-Analysis Methods with a Uniform Pipeline for Heterogeneity in microbiome studies, <https://bioc.r-universe.dev/MMUPHin/doc/manual.html#CRC_meta>). Subsequent analyses were performed on the batch-corrected abundance data (Supplementary Table S1b and S1d). All 125 samples had valid read-count statistics and were retained for downstream analysis. The mean sequencing depth was 34.19 million read pairs per sample (range: 17.43–68.86 million).

5. **Supplementary Fig S1-S4**

**5.1 Fig S1**

**Fig S1.** **Taxonomic and functional diversity across HC, ANIH, AIH and ALF groups.** (**A**) Bacterial species-level alpha diversity (left, Kruskal-Wallis with Dunn's post-hoc) and beta diversity (right, PCoA of Bray-Curtis, PERMANOVA). (**B**) Mean relative abundance of the top 20 species. (**C**) KO-level alpha and beta diversity. Box plots show median, 25th/75th percentiles, and 1.5× IQR. Ellipses, 95% confidence intervals. HC, healthy controls; ANIH, acute non-icteric hepatitis; AIH, acute icteric hepatitis; ALF, acute liver failure; KO, KEGG ortholog; PCoA, principal coordinate analysis; PERMANOVA, permutational multivariate analysis of variance.

**5.2 Fig S2.**

 

**Fig S2. Bootstrap performance of the three classification models and permutation test of the microbiome's incremental classification value**. (A) Three-model AUC comparison across 100 bootstrap iterations. Boxplots show the distribution of out-of-bag AUC values for three XGBoost classifiers: confounders only (age, sex, and BMI), microbiome only (CLR-transformed species abundances), and microbiome plus confounders. Center line, median; box limits, 25th and 75th percentiles; whiskers, 1.5× interquartile range; jittered points, individual out-of-bag AUC values. (B) Permutation test assessing the incremental classification value of the gut microbiome beyond baseline confounders. The grey histogram shows the null distribution of the incremental AUC (_Δ_AUC), defined as the AUC of the microbiome-containing model minus that of the confounders-only baseline model (age, gender, and BMI), obtained from 100 permutation iterations in which disease status labels (patient vs. healthy control) were randomly shuffled while the feature matrix was kept intact. The red vertical dashed line indicates the observed ΔAUC from the non-permuted data (observed _Δ_AUC = 0.353). The empirical *p* value was calculated as the proportion of permuted _Δ_AUC values equal to or exceeding the observed value. The observed incremental value of the microbiome markedly exceeded the null distribution (*p* < 0.01), indicating that the microbiome's contribution to disease classification is significantly greater than expected by chance and is not explained by confounding from age, gender, or BMI. AUC, area under the receiver operating characteristic curve; _Δ_AUC, incremental AUC over the confounders-only baseline.

**5.3 Fig S3.**

**Fig S3.** Abundances and detection rates of key bacterial species four groups (HC, ANIH, AIH, ALF). Left panels, bar plots of detection rates; and right panels, box plots display CLR-transformed abundance, with center lines indicating medians and box limits representing the 25th and 75th percentiles. Abundance differences, Kruskal–Wallis test; detection rate differences, chi-square test. ***p < 0.001, **p < 0.01, *p < 0.05. HC, healthy controls; ANIH, acute non-icteric hepatitis; AIH, acute icteric hepatitis; ALF, acute liver failure.

**5.4 Fig S4.**

**Fig S4.** **Functional signatures of KOs selected by at least three methods and their pathway-level associations with disease severity.** (A) Scatter plots of combined CLR abundance. Left panel, increasing subset (Spearman rho > 0, n = 217 KOs); right panel, decreasing subset (Spearman rho < 0, n = 99 KOs). Each plotted versus the residualized adjusted final score. Points colored by group. Linear regression fits with 95% confidence bands are shown. (B–C) Bootstrap distributions of Spearman rho (left) and R² (right) for the combined CLR abundance versus the residualized adjusted final score (100 iterations), shown separately for severity-increasing KOs (B) and severity-decreasing KOs (C). Red dashed line, bootstrap mean; gray dotted lines, 95% confidence interval. (D) Nine KEGG pathways significantly enriched among the selected KOs (hypergeometric test, Benjamini–Hochberg FDR < 0.05). (E) Network of significant correlations (|Spearman rho| ≥ 0.3, FDR < 0.05) between KEGG pathway abundance (computed from ≥3-method selected KOs) and nine key species. Edge width proportional to |rho|. (F) Box plots of mean CLR-transformed abundance for the significantly enriched KEGG pathways across the four groups (HC, ANIH, AIH, and ALF). Pathway abundance was computed as the mean CLR value of the KOs mapped to each pathway. Pairwise Wilcoxon rank-sum test with Benjamini–Hochberg correction: **p* < 0.05, ***p* < 0.01, ****p* < 0.001. The adjusted final score was residualized on age, sex, and BMI prior to all correlation and regression analyses. Selection required passing at least three of four criteria: Spearman |rho| > 0.3 (FDR < 0.1), Kendall |tau| > 0.3 (FDR < 0.1), bootstrap 95% CI not overlapping ±0.3, or LASSO. HC, healthy controls; ANIH, acute non-icteric hepatitis; AIH, acute icteric hepatitis; ALF, acute liver failure.

**5.5 Fig S5.**

**Fig S5. Abundance, detection rate, and correlation with disease severity of KO K08303 (lysine 2,3-aminomutase).** (**A**) Box plot of CLR-transformed abundance of K08303 across four groups (HC, ANIH, AIH, ALF). Jittered points indicate individual samples. Pairwise comparisons by Wilcoxon rank-sum test with Benjamini–Hochberg correction: ***p < 0.001, **p < 0.01. (**B**) Bar plot showing the detection rate (prevalence, %) of K08303 across four groups. (**C**) Scatter plot of K08303 CLR-transformed abundance versus the residualized adjusted final score (corrected for Age, Gender, and BMI). The black line indicates the linear regression fit with 95% confidence band (gray shading). The negative association indicates that higher K08303 abundance is significantly associated with lower disease severity, although its modest explanatory power (R² = 0.105, ~10.5% of variance explained) suggests that it captures only a limited fraction of severity variation. HC, healthy controls; ANIH, acute non-icteric hepatitis; AIH, acute icteric hepatitis; ALF, acute liver failure.

6. **Supplementary Table S1-S4**

**Supplementary Table S1 Summary of subject-level clinical data, relative abundances of key bacterial genera, and microbial functional gene (KEGG Ortholog) profiles.**

**Supplementary Table S1a** Clinical parameters and composite severity score of each subject.

**Supplementary Table S1b** Relative abundances of key bacterial genera per subject (counts per million reads).

**Supplementary Table S1c** Alpha diversity indices of the gut microbiota across HC, ANIH, AIH and ALF groups, with sample‑level measurements and group‑wise pairwise comparisons.

**Supplementary Table S1d** Relative abundances of microbial functional genes (KEGG Orthologs) per subject (counts per million reads).

**Supplementary Table S1e** Alpha diversity indices of the bacterial KOs across HC, ANIH, AIH and ALF groups, with sample‑level measurements and group‑wise pairwise comparisons.

**Supplementary Table S2 Construction and evaluation of a surrogate clinical severity index (Final Score) from routine blood biochemical and hematological parameters.**

**Supplementary Table S2a** Jonckheere–Terpstra trend test for biochemical parameters across disease severity groups.

**Supplementary Table S2b** All evaluated combinations of candidate parameters with revised composite scores (no parsimony penalty).

**Supplementary Table S2c** Sensitivity analysis of composite score weights. The original top panel (TBA, AST, PT, CHE) remained stable across most weight configurations, ranking 1st–6th among the 56 candidate panels.

**Supplementary Table S2d** Per-subject surrogate clinical severity index (Final Score and adjusted Final Score) derived from the optimal biomarker combination, with adjustment for age, sex, and BMI.

**Supplementary Table S3** **Comprehensive XGBoost classification model evaluation for discriminating hepatitis E patients from healthy controls, supporting the analyses presented in Figures 2 and S1.**

**Supplementary Table S3a** Performance metrics of the XGBoost classification model at different probability thresholds for discriminating hepatitis E patients from healthy controls.

**Supplementary Table S3b** Bootstrap-aggregated predictions and classification performance under the selected threshold (0.65).

**Supplementary Table S3c** Confounder-adjusted sensitivity analysis. Comparison of original versus confounder-adjusted (residualized) feature importance rankings, including robustness classification (Robust/Moderate/Sensitive), confounder-explained variance (R²), and rank difference (_Δ_Rank) for each species.

**Supplementary Table S3d** Adjusted feature importance stability analysis, controlling for age, sex, and BMI. For each species, the table reports mean rank, standard deviation, 95% confidence interval, and presence rate (frequency of appearance in the top features) across 100 bootstrap iterations, derived from XGBoost models that included demographic covariates.

**Supplementary Table S3e** Per-species permutation test results. For each of the 863 species, the table reports the true XGBoost Gain, mean permuted Gain, standard deviation of the null distribution, raw *p* value, Benjamini–Hochberg FDR, and significance status (FDR < 0.05 vs. NS). The four significant species (*V. atypica*, *D. longicatena*, *V. rogosae*, and *R. porci*) are highlighted.

**Supplementary Table S3f** Bootstrap-validated classification performance of confounders-only, microbiome-only, and combined models to evaluate the independent predictive value of the gut microbiome.

**Supplementary Table S4 XGBoost regression model performance, feature importance, and incremental predictive value analyses for the continuous clinical severity score, adjusted for age, sex, and BMI****, supporting the regression model presented in Figure 3.**

**Supplementary Table S4a** Bootstrap performance summary (microbiome-only, n = 100). Summary statistics of test-set R², RMSE, and Spearman correlation from 100 bootstrap iterations of the microbiome-only XGBoost regression model, adjusted for age, sex, and BMI.

**Supplementary Table S4b** Five-fold cross-validated feature importance for the regression model predicting the continuous severity score (adjusted for covariates). For each species, the table reports mean XGBoost Gain importance, standard deviation, and presence rate (frequency of selection across the five folds). Species are ranked by mean Gain.

**Supplementary Table S4c** Bootstrap feature importance (XGBoost Gain). Species-level mean XGBoost Gain, standard deviation, and presence frequency across 100 bootstrap iterations of the microbiome-only model (adjusted for covariates). Species are ranked by mean Gain.

**Supplementary Table S4d** Composite score per species. Multi-dimensional species analysis integrating XGBoost Gain, correlation with Final Score, monotonicity across disease stages, and SHAP values. The composite score is a weighted combination of standardized XGBoost, monotonicity, and correlation scores (adjusted for age, sex, and BMI).

**Supplementary Table S4e** Per-species permutation test statistics. Species-level true XGBoost Gain, mean and SD of the permutation null distribution, and permutation *p* value from 100 random permutations of microbiome features.

**Supplementary Table S4f** Five-fold cross-validated component scores (XGBoost, monotonicity, and correlation) used for composite score calculation. Composite score weights (XGBoost, monotonicity, and correlation) derived from ordinal logistic regression for each of the five CV folds.

**Supplementary Table S4g** Permutation test overall results. Observed ΔR² (microbiome − baseline), permutation *p* value, and R² values for the microbiome and baseline models from 100 permutations. Permutation testing confirmed significant incremental value over confounders (ΔR² = 0.307, *p* < 0.01).

**Supplementary Table S4h** Five-fold cross-validation regression performance and permutation-based incremental value of microbiome features for predicting the clinical severity score.

**Supplementary Table S5 Bootstrap validation and functional gene (KO) association statistics for microbiome regression, combined abundance modeling, and species–gene correlation analyses, supporting the regression model presented in Figures 4 and S3.**

**Supplementary Table S5a** Feature selection results across the four independent methods (Spearman, Kendall, bootstrap, and LASSO). Per-KO pass/fail status under each criterion, the 16-KO four-method intersection, and the 316-KO set selected by at least three methods; LASSO cross-validation parameters (lambda.min = 3.61, 33 KOs; lambda.1se = 6.61, 12 KOs) are included.

**Supplementary Table S5b** Bootstrap resampling distribution of correlation and regression statistics for the four-method intersection KO set, split into severity-increasing and severity-decreasing subsets by direction of association with the residualized adjusted final score. For each of 100 iterations, samples were drawn with replacement from the full dataset; combined CLR abundance was computed as the mean of the 4-method selected KOs within each subset, and both Spearman correlation and linear R² against the residualized adjusted final score were recorded. These raw values underpin the histogram visualizations and 95% confidence intervals reported in Figure 4D and E.

**Supplementary Table S5c** Pairwise correlation statistics of four-method selected KOs and nine key bacterial species based on CLR-transformed abundance.

**Supplementary Table S5d** Bootstrap resampling distribution of correlation and regression statistics for the KO set selected by at least three methods, split into severity-increasing and severity-decreasing subsets by direction of association with the residualized adjusted final score. For each of 100 iterations, samples were drawn with replacement from the full dataset; combined CLR abundance was computed as the mean abundance of the selected KOs within each subset, and both Spearman correlation and linear R² against the residualized adjusted final score were recorded. These raw values underpin the histogram visualizations and 95% confidence intervals reported in Figure S3B and C.

**Supplementary Table S5e** KEGG pathway enrichment results for the KO set selected by at least three methods. Pathway hierarchy, KO counts, gene ratios, enrichment statistics, and FDR-adjusted q-values are shown for significantly enriched pathways (q < 0.05), reported in Figure S3D.
